# Cytoskeleton Elements Contribute to Prion Peptide-Induced Endothelial Barrier Breakdown in a Blood–Brain Barrier In Vitro System

**DOI:** 10.3390/ijms232012126

**Published:** 2022-10-12

**Authors:** Itzik Cooper, Katayun Cohen-Kashi Malina, Yishai Levin, Alexandra Gabashvili, Boaz Mohar, Alfredo Cagnotto, Mario Salmona, Vivian I. Teichberg

**Affiliations:** 1 The Joseph Sagol Neuroscience Center, Sheba Medical Center, Tel Hashomer, Ramat Gan 52621, Israel; 2 School of Psychology, Reichman University, Herzliya 46150, Israel; 3 Department of Neurobiology, Weizmann Institute of Science, Rehovot 76100, Israel; 4 The Nancy and Stephen Grand Israel National Center for Personalized Medicine, de Botton Institute for Protein Profiling, Weizmann Institute of Science, Rehovot 76100, Israel; 5 Department of Molecular Biochemistry and Pharmacology, IRCCS-Istituto di Ricerche Farmacologiche “Mario Negri”, Via Mario Negri 2, 20156 Milano, Italy

**Keywords:** blood–brain barrier, PrP 106-126, cytoskeleton, brain endothelial cells, LC-MS

## Abstract

The mechanisms involved in the interaction of PrP 106-126, a peptide corresponding to the prion protein amyloidogenic region, with the blood–brain barrier (BBB) were studied. PrP 106-126 treatment that was previously shown to impair BBB function, reduced cAMP levels in cultured brain endothelial cells, increased nitric oxide (NO) levels, and changed the activation mode of the small GTPases Rac1 (inactivation) and RhoA (activation). The latter are well established regulators of endothelial barrier properties that act via cytoskeletal elements. Indeed, liquid chromatography-mass spectrometry (LC-MS)-based proteomic profiling study revealed extensive changes in expression of cytoskeleton-related proteins. These results shed light on the nature of the interaction between the prion peptide PrP 106-126 and the BBB and emphasize the importance of the cytoskeleton in endothelium response to prion- induced stress.

## 1. Introduction

The blood–brain barrier (BBB) is a dynamic barrier providing homeostasis, protection, and nutrition for the brain [1,2,3]. It maintains the chemical composition of the neuronal “milieu”, which is required for proper functioning of the brain [4]. On the other hand, it filters harmful compounds from the brain back to the bloodstream and shields the brain from toxic substances in the blood [5].

The BBB is formed by brain endothelial cells (BEC) lining the cerebral microvasculature, together with perivascular elements such as closely associated astrocytic end-feet processes, perivascular neurons and pericytes [6,7]. Two tightly controlled pathways for molecules and cells to cross the BBB exist: (a) A transcellular pathway across the membranes of individual cells, mediated mostly by channels, carriers, pumps and vesicles and (b) A very restricted paracellular pathway through the intercellular junctions mediated by the inter-endothelial tight junction (TJ) [8] which are linked to the actin cytoskeleton in multi-protein complexes [9].

Prion diseases, also known as transmissible spongiform encephalopathies, are human and animal diseases characterized by progressive neuronal loss, which is often accompanied by a spongiform brain alteration and the deposition of amyloid fibrils. These diseases appear in sporadic, familial, and infectiously acquired forms, and are invariably fatal to the host [10].

Although very little is known about prions association with the BBB, there is evidence linking prions to functional impairment of the BBB [11,12,13,14,15,16,17,18]. Moreover, Banks et al. [11,12] have shown that both the scrapie prion protein, PrPSc, and the cellular prion protein, PrPC, have the ability to cross the BBB in-vivo. In further support to these notions, we have shown [14,19] that the prion peptide PrP 106-126 is toxic to BEC and causes the BEC to undergo a coordinated remodeling of the TJ and an expansion of the cell surface.

The PrP 106-126 peptide corresponds to the 106-126 amyloidogenic region of the cellular human prion protein (PrPC) and its biochemical properties resemble the infectious form of the prion protein [20]. Previous observations have shown that the peptide fragment PrP 106-126, which is reported to account for the neurodegeneration seen in prion disease, can also be taken up by glial cells [21,22] and causes microglia activation and differentially affects their cytokine secretion [5]. Moreover, the PrP 106-126 sequence is present in all abnormal prion isoforms accumulated in brains of prion disease patients [23,24] making it highly relevant for prion-related research.

As one of our major previous findings [14] was that BEC increase their cell area upon exposure to the PrP 106-126, and that TJ and adherence junctions (AJ) expression is altered, we wished to examine whether actin filaments and other cytoskeleton elements are involved in these changes. Therefore, we tested here whether the PrP 106-126 influences the levels of cAMP, an established modulator of barrier function at the periphery as well as in the CNS [25,26,27,28], and the activation state of small guanosine triphosphatases (GTPases), which were shown to control barrier properties via a reorganization of the junction-associated cortical actin cytoskeleton. In that context, it should be mentioned that studies with several barrier-disruptive components (usually, inflammatory agents) found them to increase the barrier permeability by a reduced formation of cAMP, and inactivation of the small GTPase Rac1 along with the activation of RhoA [29]. A proteomic approach (LC-MS) revealed that a substantial portion of the altered proteins were cytoskeleton-related, emphasizing its importance in BEC’s response to PrP 106-126-induced damage.

## 2. Results

### 2.1. Brain Capillary Endothelial Cells

**Figure 1:**
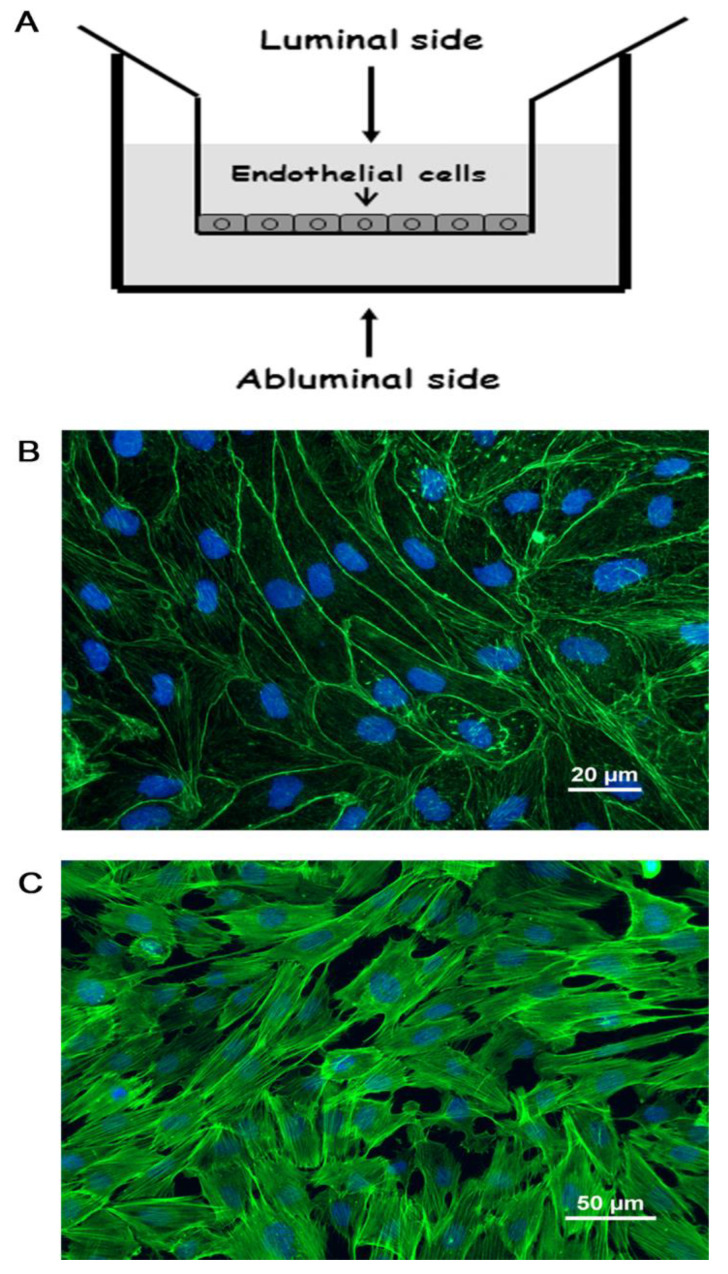
Brain capillary endothelial cells. (**A**) Schematic drawing of the in-vitro BBB culture system used. PBEC were cultured on collagen coated Transwell inserts to gain typical BBB-like characteristics. (**B**,**C**) Immunocytochemistry of the two types of brain endothelial cells used: PBEC (**B**) and the human cell line hCMEC/D3 (**C**). Cells were stained for F-actin using Alexa Fluor 488-conjugated phalloidin (green). Nuclei were counterstained with Hoechst.

In this study, we used both human and porcine brain endothelial cells (PBEC). For the in-vitro BBB model, we used a primary cell culture prepared from porcine brains that very closely mimics the in-vivo characteristics of the BBB [30,31]. In addition, we used the human cell line hCMEC/D3, which are well characterized as a human in vitro model [32]. The human cell line was used at sub-confluence to study the response of signaling molecules after PrP 106-126 exposure. The actin fibers localization of these two types of cells under normal conditions, and the scheme of the in-vitro BBB model are displayed in Figure 1.

### 2.2. PrP 106-126 Alters cAMP and Nitric Oxide Levels in Brain Endothelial Cells

**Figure 2:**
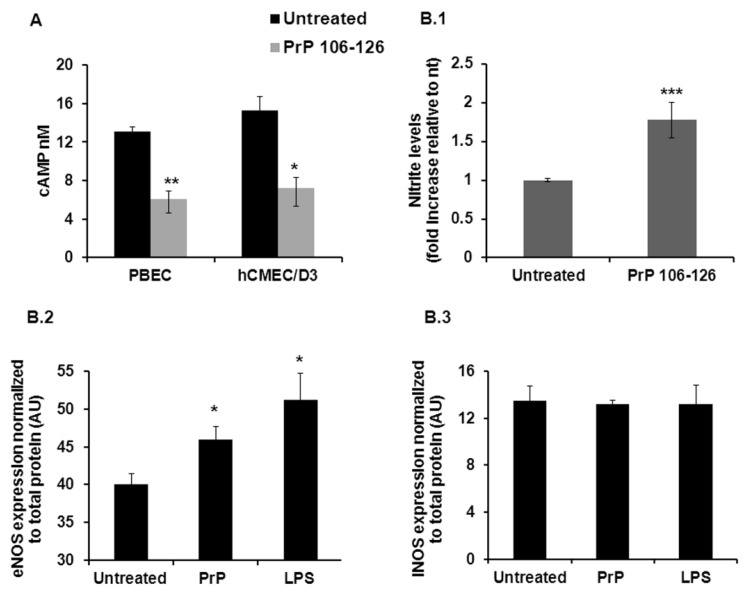
cAMP, NO and NOS levels in PBEC after PrP 106-126 treatment. (**A**) cAMP levels decreased by more than 50% after 2 h treatment with PrP 106-126 in both PBEC and human brain endothelial cell line (*n* = 3). Results are expressed as the mean ± SEM. (**B.1–B.3**) NO levels increased in the cell medium after exposure to PrP 106-126 (**B.1**); NO levels were measured in the supernatants of PBEC treated with PrP 106-126 or non-treated cells using a Griess colorimetric assay (*n* = 14–29). The expression level of eNOS (**B.2**) but not of iNOS (**B.3**) was elevated after exposure to PrP 106-126 (2 h treatment, expression levels were measured using in-cell western blot (protocol is described in Section 4), *n* = 3–4. LPS served as positive control [37]. * *p* < 0.05, ** *p* < 0.01, *** *p* < 0.001 compared with untreated. nt; not treated, PrP; PrP 106-126.

Adenylate cyclase-stimulating agents are well known to reduce endothelial permeability [33] and increased intracellular cAMP can strengthen endothelial barrier functions in the periphery as well as at the BBB [26,27,34,35]. Since we previously demonstrated that the prion peptide, PrP 106-126, causes significant morphological alterations in BEC [14], we examined here whether changes in the intracellular levels of cAMP might be involved in these processes. We found that PrP 106-126 indeed reduced cAMP levels both in PBEC and in the human brain endothelial cell line hCMEC/D3 to a very similar extent (54% and 53% reduction respectively) (Figure 2A).

cAMP is a known regulator of nitric oxide synthases (NOS), enzymes which catalyze the production of nitric oxide (NO) from L-arginine. NO is a key signaling molecule in the nervous system as well as in the regulation of vascular properties [36]. We thus examined whether PrP 106-126 affects the production levels of NO. We found that after PBEC were treated with PrP 106-126, NO levels were increased by 78%. This increase was accompanied by an elevation of eNOS (endothelial nitric oxide synthase) but not of iNOS (inducible nitric oxide synthase) protein expression levels (Figure 2(B.1–B.3)).

### 2.3. PrP 106-126 Modulates the Activation State of the Small GTPases Rac1 and RhoA

**Figure 3:**
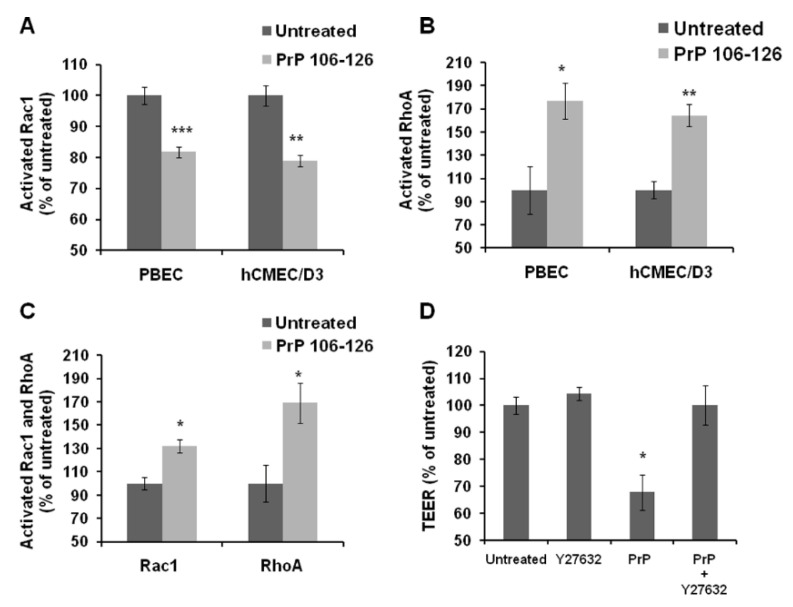
Involvement of small GTPases in PrP-induced damage to the BBB. (**A**) Rac1 in PBEC and in hCMEC/D3 was inactivated after 2 h exposure to 100 µM PrP 106-126. Activation of Rac1 was measured by using G-Lisa colorimetric assay (*n* = 3 for each cell type). (**B**) RhoA in PBEC and in hCMEC/D3 was activated after 2 h exposure to 100 µM PrP 106-126. Activation of RhoA was measured by using G-Lisa assay (*n* = 3 for each cell type). (**C**) RhoA was still activated after 24 h exposure to 100 µM PrP 106-126 in PBEC while Rac1 changed its activation mode from inhibition (**A**) to activation (*n* = 3 for each cell type). (**D**) Inhibition of Rho kinase by 50 µM Y27632 together with 100 µM PrP 106-126 prevented the decrease in TEER observed when treating only with PrP 106-126 for 24 h in an in-vitro BBB model (*n* = 3–5 Transwell inserts). Results are expressed as the mean ± SEM. * *p* < 0.05, ** *p* < 0.01, *** *p* < 0.001 compared with untreated. PrP; PrP 106-126.

Reduced formation of cAMP can lead to the activation or inactivation of members of the Rho GTPases family. These proteins are involved in cell adhesion and cytoskeletal dynamics and serve as important regulators of endothelial barrier functions. More particularly, in most cases of barrier disruption, the small GTPase Rac1 is inactivated while RhoA is activated [29]. Decrease of cAMP leading to Rac1 inactivation was found to be the mechanism underlying endothelial barrier breakdown in response to the inflammatory mediators TNFα and LPS [38,39]. In addition to their involvement in cytoskeleton dynamics, Rho GTPases may modify endothelial barrier functions directly by altering the cells junctional proteins which we have previously shown to be affected by PrP 106-126 treatment [14]. In this previous publication we have specifically shown that PrP 106-126 decreased the expression levels of claudin-5, occludin and Ve-cadherin but had no effect on ZO-1. In line with these published results, we found that following 2 h treatment with PrP 106-126, Rac1 was inactivated while RhoA was activated (Figure 3A,B) both in PBEC and in human brain endothelial cells. After 24 h exposure to PrP 106-126, RhoA was still activated while Rac1 activation state changed from non-active (after 2 h) to active in comparison to control cells (Figure 3C).

Investigating whether the inhibition of the RhoA pathway could have beneficial effects on barrier properties, we used Y27632, a Rho kinase inhibitor (which is the enzyme mediating the RhoA destabilizing effects in endothelial barriers). Addition of Y27632 to the medium together with PrP 106-126, prevented the observed decrease in TEER following PrP 106-126 application (Figure 3D). These data suggest that the small GTPases Rac1 and RhoA participate in the mechanism of the PrP-induced damage to brain endothelial cells, where Rac1 is inactivated, while the RhoA pathway is activated by PrP 106-126.

### 2.4. Stress Fibers Formation and Cortactin Reduction in Brain Endothelial Cells Exposed to PrP 106-126

**Figure 4:**
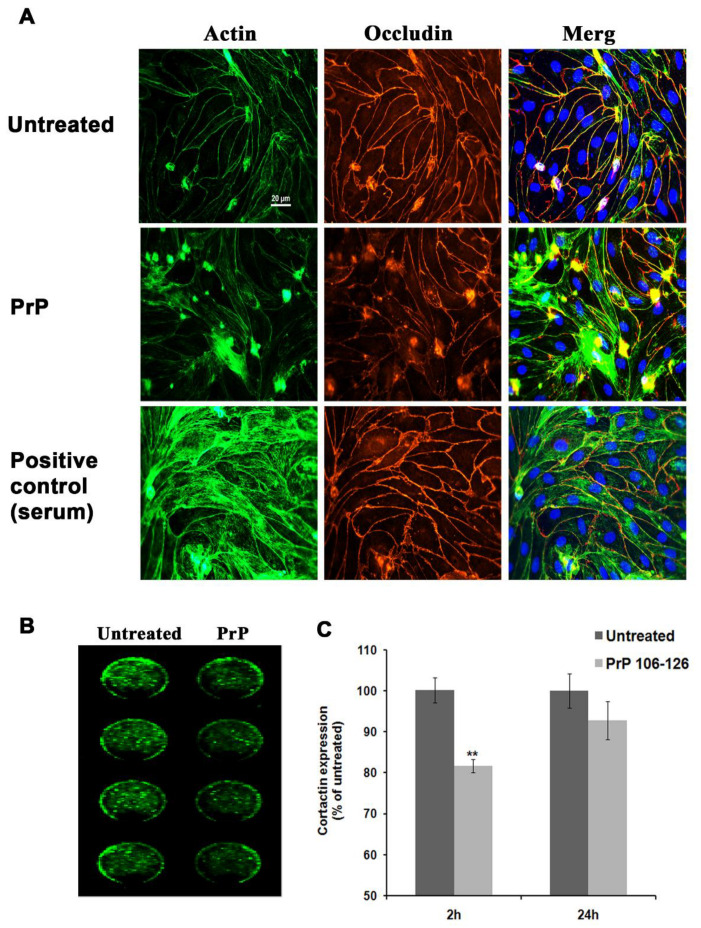
Stress fibers formation and cortactin expression levels in brain endothelial cells exposed to PrP 106-126. (**A**) Stress fibers formation after PrP 106-126 treatment is shown in PFA fixed PBEC monolayers after 24 h exposure to 100 µM PrP 106-126 in assay medium or plating medium which contains 10% serum for positive control. (**B**) In-cell western blot showing a reduction in cortactin protein levels after 2 h treatment with 100 µM PrP 106-126. An image from the Odyssey infra-red detector is shown. (**C**) Quantification of B. After 2 h exposure to PrP 106-126 a statistically significant difference between the treated and untreated groups was found. The reduction after 24 h was not statistically significant. Results are expressed as the mean ± SEM (*n* = 4). ** *p* < 0.01, compared with untreated cells normalized to total cell proteins staining (Section 4).

Since small GTPases modulate endothelial barrier properties by regulating cytoskeletal dynamics, we next examined whether PrP 106-126 affects the actin filaments structure and the actin-binding protein cortactin which is known to accumulate at cell borders under conditions of improved barrier properties [38,40,41,42,43]. To that end, we inspected the morphology of PBEC monolayers immunostained with fluorescence-labeled phalloidin which binds very strongly to F-actin and the TJ protein occludin. Although we could not detect any typical gap formations which are often reported when treating endothelial or epithelial monolayers with barrier-disruptive agents (Figure 4A), stress fiber induction in the cell cytoplasm could be observed, at least in some areas of the monolayer, in parallel with a reduced immunostaining of occludin. Figure 4A shows that in untreated endothelial monolayer, actin is mostly distributed at the cell borders in a very orderly and tight manner. The formation of stress fibers is very obvious in serum-treated cells (Figure 4A, lower panels), serving as positive control (serum factors induce stress fibers via small GTPases activation [44]).

In order to monitor cortactin expression levels in PBEC monolayers, we used the in-cell western blot method. After 2 h treatment with PrP 106-126, the total protein levels of cortactin were significantly reduced by 20%. However, following 24 h treatment the cortactin expression levels recovered almost fully (Figure 4B,C). 

### 2.5. PrP 106-126 Increases Cytoskeleton-Related Proteins Expression in Brain Endothelial Cells

Inspired by the above results and considering our previous study [14] in which we have shown that BEC exposed to PrP 106-126 responded to this stress by enlarging their surface area territory, compensating for the cell lost and therefore strongly suggesting that cytoskeletal elements are involved in the PrP 106-126- induced damage, we used global proteomic analysis to test the identity of the proteins which were modified after exposure of PBEC monolayer 24 h to PrP 106-126 in comparison to untreated cells.

**Figure 5:**
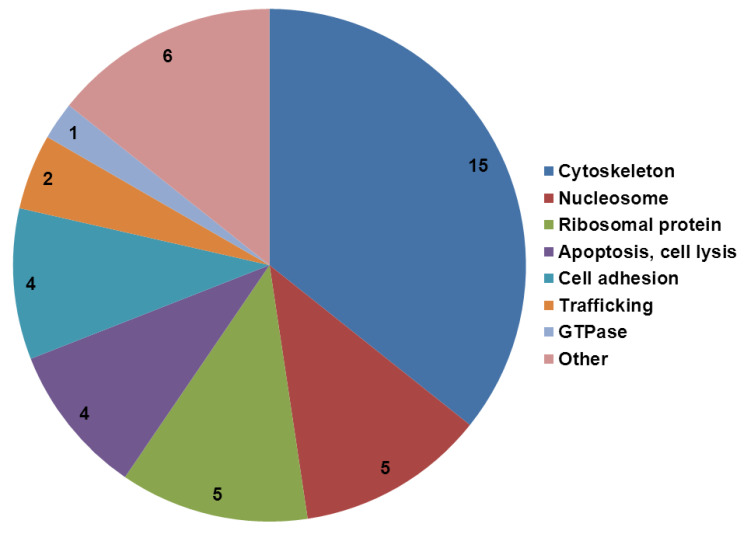
Distribution and characterization of the statistically significant modified proteins in PBEC exposed to PrP 106-126. PBEC were treated with 100 µM PrP 106-126 or with vehicle for 24 h. Proteins expression levels were analyzed using LC-MS (Section 4). Some of the proteins have overlapping functions.

The expression levels of 34 out of 628 proteins identified showed statistically significant differential expression; 44% of them were directly related to cytoskeleton functions (Tables S1 and S2 in the supporting information), all showing increase in the expression levels. Using targeted LC-MS analysis we were able to confirm the results in 10 proteins tested and found that all were indeed up regulated (Table S3). The other significantly altered proteins could be divided into functional groups related to nucleosome, ribosomal proteins, apoptosis/cell lysis and trafficking (Figure 5).

## 3. Discussion

**Figure 6:**
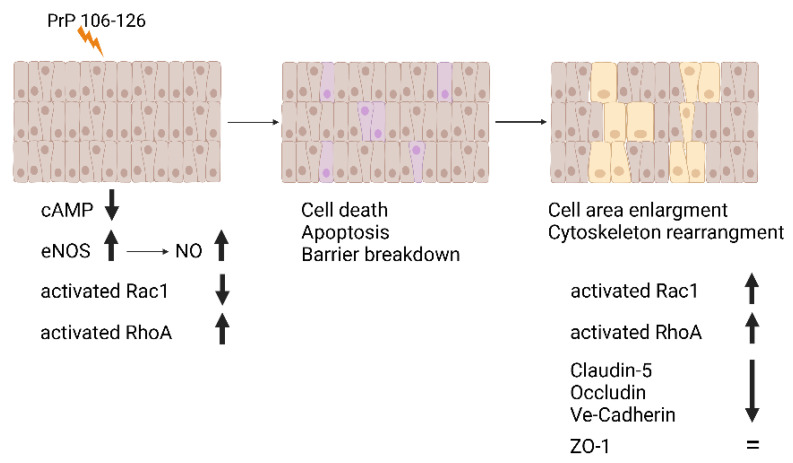
Schematic illustration of PrP 106-126-induced damage and endothelial compensating response. PrP 106-126 exposure to BEC results in cell death and apoptosis, reduction in TJ and AJ and partial loss of barrier function which are compensated by an enlargement of the endothelial cell territory rather than by cell replication [14]. This remodeling of the intercellular interactions of the remaining cells is mediated by activation and de-activation of the small GTPases Rac1 and RhoA which affect barrier integrity (Figure 3). Reduction in cAMP and elevation of eNOS and NO occur after exposure to PrP 106-126; The exact mechanism beyond the effects of these molecules on the robust changes observed in cytoskeleton proteins (Figure 4 and Figure 5 and Supplementary Tables S1–S3) remains to be elucidated.

In this study, we investigated the biochemical consequences of the interaction between the PrP 106-126 peptide and brain endothelial cells. As we have previously shown [14], this interaction compromises barrier integrity by reducing TEER, redistributing TJ and AJ, initiating programmed cell death and causing a cell territory expansion to compensate for the loss of cells in the endothelial monolayer (Figure 6).

GTPases of the Rho family are known to be involved in the regulation of cell-cell junctions and cytoskeletal dynamics, thus making them important regulators of endothelial barrier functions [45]. The inflammatory mediators LPS and TNF-α are known to induce endothelial barrier breakdown by decreasing cAMP levels which lead to Rac1 inactivation, that precede RhoA activation [38,39]. Interestingly, we found that PrP 106-126 acts in a similar way to these inflammatory agents by reducing cAMP levels in brain endothelial cells (both human and porcine, Figure 2A). The pattern of Rac1 inactivation and RhoA activation mentioned above was also found when brain endothelial cells were exposed to the prion peptide (Figure 3A,B). Surprisingly, after 24 h Rac1, which was inactivated after 2 h of exposure, became activated. Possibly this reflects the barrier response in an attempt to maintain its integrity while coping with the PrP-induced damage. In support to this interpretation, we previously reported that the exposure of an in-vitro BBB to PrP 106-126, although caused a reduction in TEER, did not cause a complete breakdown of the barrier [14]. Figure 3D shows that inhibition of Rho kinase, a RhoA effector protein, can restore the PrP-induced barrier breakdown suggesting a crucial role for this pathway in the PrP-BBB interaction. These results are strengthened by the observations of Schreibelt et al. [46] who showed that reactive oxygen species (ROS) alter BBB integrity via signaling pathways, including RhoA, PI3 kinase and protein kinase B and that inhibition of these pathways prevented the ROS-induced damage.

Nitric oxide (NO) is a key signaling molecule in the nervous system as well as in the regulation of vascular properties exerting both beneficial and detrimental actions [36]. We observed that NO levels are influenced by PrP 106-126 treatment. More specifically, NO levels increased by 78% following PBEC treatment with PrP 106-126. This increase was paralleled with an elevation in protein levels of eNOS but not in iNOS (Figure 2(B.2,B.3)). The iNOS isoform is usually induced in the body during inflammation by the presence of certain inflammatory cytokines or bacterial products [47]. The relatively moderate increase in NO that we found could be attributed to the action of eNOS, which is constitutively expressed, in contrast to iNOS expression, and which results in more robust NO production. These results coincide with the findings of Park et al. [48] that eNOS expression levels are elevated in prion disease.

Since cAMP is known to be a regulator of NOS expression [36] it is reasonable to assume that the changes in cAMP levels can contribute to the changes in NO via eNOS expression. Although a further investigation is needed, one can speculate that the observed eNOS mediated increase in NO levels along with the decrease in cAMP levels can at least in part contribute to the damaging effects caused by the prion peptide.

Given that one of our major earlier findings was that PBEC increase their cell area upon exposure to the PrP 106-126, we also examined whether actin filaments as well as other cytoskeleton elements may be involved in these changes. We detected stress fiber formation upon exposure to PrP 106-126 (Figure 4A). This was not as robust as the impact of a PrP 106-126 treatment on TJ and AJ of PBEC [14], suggesting that the loss of the intercellular adhesion via TJ and AJ might be more crucial to barrier malfunction. When examining the actin-binding protein cortactin which accumulates at cell borders under conditions of improved barrier properties [41,42,49] and participates in processes of bacterial and immune cell penetration through endothelial cells [50,51] via GTPases-mediated pathways, no changes in protein distribution in response to PrP 106-126 could be detected, but a decrease in total protein expression was found after 2 h treatment (Figure 4B,C). These findings may implicate the involvement of cortactin in PrP-induced processes at the BBB. Since the expression levels of cortactin were restored almost completely after 24 h, we speculate that as in the case of Rac1 (see above), this may be a part of the BBB response to maintain integrity. Further experiments however are needed to elucidate the exact mechanisms and relationships between the different cytoskeleton components involved.

The data obtained from the LC-MS experiments (Figure 5 and Tables S1–S3 in the supporting information) not only confirm the robust participation of cytoskeleton elements in the response of endothelial cells to the peptide, it clearly demonstrates that the nature of the response in the case of the endothelial cells lining the BBB is mainly in the morphology of the cells as 44% of the modified proteins were cytoskeleton-related proteins. These observations coincide with our previous ones [14] where we have shown that PrP 106-126 caused cell death in PBEC monolayer but the barrier, although weakened, remained complete by using a unique mechanism of expanding the cell area spreading through the gaps formed by the dying cells (Figure 6).

As far as we know, in the only other “omic” approach using PrP 106-126, Martinez et al. [52] treated SH-SY5Y neuroblastoma cells with PrP 106-126 for 6 h and found, using a DNA microarray, several deregulated genes, mainly involved in biosynthesis, transport, folding and catabolism of proteins, and in the regulation of cell cycle. They stress that this prion fragment only alters a relatively low number of cytoskeletal-related genes in the SH-SY5Y neuroblastoma cells. We believe that the different distribution of modified gene/proteins between the two types of cells, i.e., neurons and endothelial cells of the BBB, reflects well their function in the brain; the endothelial cells lining the BBB have a barrier function and as such it is very reasonable that barrier functions will need to be conserved after an insult. Thereby, active morphological changes are necessary to maintain the barrier as intact as possible.

The other significantly altered proteins obtained in the proteomic analysis could be divided into functional groups related to nucleosome (15%), ribosomal proteins (15%), apoptosis/cell lysis (12%), cell adhesion (12%), and trafficking (6%). Some of these changes are clearly related to the cells response in the direction of new genes/proteins synthesis (nucleosome, ribosome), which can be related to the elevation in cytoskeleton proteins as part of the coping mechanism towards the PrP-induced stress.

In summary, our results suggest that the cellular response of BEC lining the BBB to the prion fragment PrP-106-126 is dominated by cytoskeleton elements which possibly enable the continuous well-functioning of the barrier (at least to some extent) despite the damage induced.

## 4. Materials and Methods

### 4.1. Ethics

All procedures involving animals were reviewed and approved by the Weizmann Institutional Animals Care Committee (approval number 04520810-2, “Elaboration of an in vitro blood brain barrier”).

### 4.2. Materials

Mouse anti-occludin and rabbit anti-ZO-1 were purchased from Invitrogen. Rabbit anti-cortactin antibody was from Abcam. Rabbit anti-iNOS and eNOS were obtained from Santa Cruz Biotechnology, (Santa Cruz, CA, USA). Mouse anti-prion protein (12F10), for detection of the cellular prion protein was obtained from Cayman (Cayman Chemical, Ann Arbor, MI, USA). Alexa Fluor 488-conjugated phalloidin was purchased from Molecular Probes. Cy and Alexa Fluor-conjugated secondary antibodies were acquired from Jackson Immunoresearch and Molecular Probes, respectively, and used for immunocytochemistry. IR-conjugated secondary antibodies were purchased from LI-COR Biosciences and were used for the in-cell western blot experiments. Unless otherwise mentioned, all other materials used were purchased from Sigma-Aldrich Israel Ltd. (Rehovot, Israel).

### 4.3. Media

‘Plating medium’ was composed as follows: 10% newborn calf serum, 2 mM L-glutamine, penicillin/streptomycin (100 units/mL and 0.1 mg/mL respectively), 0.1 mg/mL gentamycin (Biological industries, Kibbutz Beit-Haemek, Israel) in Earl’s Medium 199 (Sigma). ‘Assay medium’ contained: 2 mM L-glutamine, penicillin/streptomycin (100 units/mL and 0.1 mg/mL respectively), 0.1 mg/mL gentamycin, 550 nM hydrocortisone (Sigma) in Dulbecco-modified Earl’s medium (DMEM) diluted 1:1 in Ham’s F12 medium (Biological industries, Israel). For growing hCMEC/D3 (brain endothelial cell line) EGM-1 medium was used: EGM-2 medium (Lonza) supplemented with FBS, bFGF, gentamycin, ascorbic acid and hydrocortisone according to the manufacture instructions (EGM-2 SingleQuots, LONZA).

### 4.4. Peptides

PrP 106-126, derived from amino residues 106–126 of the human prion protein sequence (sequence: KTNMKHMAGAAAAGAVVGGLG) was synthesized as described before [53]. For assessing the BBB response, brain endothelial cells were treated with PrP 106-126 freshly made in double distilled water to 5.23 mM stock solution. This stock was immediately diluted in the treatment medium to the desired final concentrations and added to the cell culture.

### 4.5. Cells

Primary cultures of endothelial cells were isolated from freshly collected porcine brain as previously described [31]. The purity of the culture was confirmed by specific staining for Von-Willebrand factor [30]. PBEC were either seeded as monolayer on 24/96 well plates or used for in vitro model of the BBB. The immortalized human brain endothelial cells line, hCMEC/D3, was the kind gift Professor Babette B. Weksler and was cultured according to Lonza (Basel, Switzerland) instructions for working with EGM^®^-2 medium. When studying signaling events (cAMP measurements and GTPases activity assays) the PBEC and hCMEC/D3 were used at sub-confluence, otherwise PBEC were used at full confluence.

### 4.6. In-Vitro BBB Model and Transendothelial Electrical Resistance Measurements

The properties of the in vitro BBB model were previously described [30]. Shortly, PBEC were seeded at a density of 100,000 PBEC/cm^2^ on microporous membrane of a Transwell insert (Corning Costar, Acton, MA, USA) placed into the 12 well plates. The cells were cultured in plating medium for up to 3 days until they reached confluency. Then, the medium was replaced with a serum-free medium (assay medium) for an additional 24 h. The integrity of the in vitro BBB model was determined by measuring the trans-endothelial electrical resistance (TEER) of the cellular barrier formed by the PBEC monolayers. The TEER reflects the impedance to the passage of small ions through the physiological barrier and is widely recognized as one of the most accurate and sensitive measures of BBB integrity. A decrease in TEER reflects an increase in permeability and a loss of barrier function.

TEER of the filter insert was recorded using an Endohm chamber connected to an EVOM resistance meter (World Precision Inst., Inc., Sarasota, FL, USA). The TEER of each filter insert was calculated by subtracting the TEER of the microporous membrane without PBEC and is reported as Ωcm^2^. For testing its effects on TEER, PrP 106-126 was diluted in assay medium at the desired concentration and added to the luminal side of the filters. When testing the Rho kinase inhibitor, 50 µM of Y27632 (Cayman Chemical, Ann Arbor MI, USA) were applied to the luminal side together with 100 µM PrP 106-126 and the TEER were monitored. A thorough investigation about the specific effects of PrP 106-126 (including the lack of effects of the scrambled peptide) on barrier integrity as reflected by TEER kinetics was previously published by us [14].

### 4.7. cAMP Determination

PBEC and hCMEC/D3 were grown on collagen-coated 96-well plates in plating medium and EGM-1 medium respectively. The media were removed and replaced with assay medium or fresh EGM-1 medium containing 100 µM PrP 106-126 peptide and incubated at 37 °C for the indicated time. cAMP content was determined with cAMP-Glo^TM^ luminescence Assay (Promega, cat # V1501) according to the manufacturer instructions.

### 4.8. Nitrite Measurements

NO production was determined by the Griess assay. This method is based on the measurement of nitrite, a stable end product of NO and oxygen. Supernatants from PrP 106-126-treated PBEC (1–6 h) were collected and 100 µL were mixed with equal volume of Griess reagent (0.1% *N*-[1-naphtyl]ethylenediamine dihydrochloride, 1% sulphanilamide in hydrochloric acid) for 1 h at room temperature. Absorbance was measured at 550 nm with a microplate reader. The nitrite concentration was determined from a sodium nitrite standard curve ranging from 0 to 12 µM. All experiments were performed in duplicate, in 7–14 independent experiments. The fold increase of nitrite production from the PrP 106-126 treated cells was calculated relatively to untreated cells in each experiment.

### 4.9. eNOS, iNOS and Cortactin Expression

The expression levels of eNOS and iNOS enzymes as well as for cortactin was determined with an in-cell western blot assay as previously described [30] with some modifications. Shortly, PBEC were grown on collagen-coated 96-well plates in plating medium. The cells were treated with PrP 106-126 (100 µM) and with lipopolysaccharide (LPS) as positive control (at 20 µg/mL, derived from Salmonella typhimurium). The treatments were carried out for the time indicated in the text. The cells were then fixed with 4% paraformaldehyde in PBS for 10 min. For normalization of protein expression levels, we used a total protein labeling protocol in which the cells are stained for 20 min with IRDye NHS800CW (LI-COR Biosciences, Lincoln, NE, USA), which covalently label proteins in cells by coupling to free amino groups to form a stable conjugate. After this step, the cells were washed and permealized with 0.1% Triton/PBS followed by 1 h incubation in blocking buffer (LI-COR Biosciences). Then, the cells were incubated with the primary antibodies at 1:40 dilutions in blocking buffer. Following several washing steps, the cells were probed with appropriate secondary antibodies (IRdye 680CW donkey anti-rabbit) at a concentration of 1:200 (1 h RT). The intensity of each well was quantified at both the 680 nm (for iNOS, eNOS or cortactin) and 800 nm (for total protein) channels using the Odyssey analysis software version 3.0.

### 4.10. Rac1 and RhoA Activation Assay

Cells were grown and treated in 24-well plates as indicated in the text. For measurement of Rac1 and RhoA activation, the Rac1 and RhoA G-Lisa Activation Assays Biochem Kits (Cytoskeleton, Denver, CO, USA) were used according to the manufacturer’s recommendations. The signal was measured at 490 nm using a microplate spectrophotometer.

### 4.11. Immunocytochemistry

The purity (>99%) of the primary cultures was assessed using specific endothelial cell marker (Von Willebrand factor, [30]). PBEC were grown at a density of 250,000 cells/24-well on rat tail collagen coated slides (27 µg/mL, 180 µL/slide in 24-well plate, dried for 24 h at room temperature) or on Transwell inserts as indicated above. After treatments, the cells were fixed with 4% paraformaldehyde (PFA) for 10 min at room temperature and exposed to blocking solution (20% donkey serum/0.1% triton/Phosphate-buffered saline (PBS)) for 2 h. The PBEC were then incubated with mouse anti-occludin, rabbit anti ZO-1 or mouse anti prion antibodies (1:200, overnight, 4 °C), washed with PBS and stained with Cy3-labeled secondary antibodies (1:200, 1 h, room temperature). Actin filaments were stained with Alexa Fluor 488-conjugated phalloidin (3 µL/insert, incubated together with the secondary antibody). Nuclei were counterstained with Hoechst (20 s). Images were taken on Nikon *Eclipse 80i* fluorescence microscope.

### 4.12. Liquid Chromatography-Mass Spectrometry (LC-MS)

#### 4.12.1. Proteomic Analysis

PBEC were grown to confluent and treated with 100 µM PrP 106-126 for 24 h as previously described [14]. Proteins were extracted from total cell lysate and the concentration was determined using standard bicinchoninic acid (BCA) assay. Proteins from each sample were reduced using 5 mM dithiothreitol (Sigma) in 60 °C for 30 min. This was followed by alkylation using 10 mM iodoacetamide for 30 min in room temperature, in the dark. Trypsin was then added at a ratio of 50:1 (protein:trypsin) and samples were incubated overnight at 37 °C. Digestion was stopped by addition of 1%TFA.

#### 4.12.2. Shotgun Proteomics

Digested proteins (15 μg) from each sample were analyzed by nano-Ultra Performance Liquid Chromatography (2D nanoAcquity; Waters) in high-pH/low-pH reversed phase (RP) 2 dimensional liquid chromatography mode, coupled to high resolution, high mass accuracy mass spectrometry (Synapt G2 HDMS, Waters). The quadrupole ion mobility time-of-flight mass spectrometer was tuned to 20,000 mass resolution (full width at half height). Data were acquired in HDMS^E^ positive ion mode in data independent acquisition (for further details see Supplemental Methods).

Raw data were imported into Rosetta Elucidator^®^ System, version 3.3 (Rosetta Biosoftware, Seattle, WA, USA). Elucidator was used for alignment of raw MS1 data in RT and m/z dimensions. Alignment, feature identification, and extraction across the chromatographic time window were performed (as described in http://onlinelibrary.wiley.com/doi/10.1002/pmic.201000661/full (1 December 2011)). Protein abundance was calculated based on the three most abundant peptides per proteins (http://www.mcponline.org/content/5/1/144 (1 December 2011)).

#### 4.12.3. Targeted Proteomics—Selective Reaction Monitoring

In order to validate some of the findings in the shotgun proteomics study we used stable isotope dilution mass spectrometry using a tandem quadrupole instrument. A total of 26 peptides from 10 proteins were synthesized with heavy stable isotopes (JPT Technologies, Berlin, Germany). Heavy labels included U-^13^C_6_; U-^15^N_4_ for peptides terminating with Arg and U-^13^C_6_; U-^15^N_2_ for Lys. Samples were run blindly and randomly using a nanoLC coupled to a tandem quadrupole MS, operated in SRM mode (see Supplementary Information for further details).

### 4.13. Statistical Analysis

Statistical analysis was performed using the Prism software. Data are presented as means ± standard error or standard deviation. Differences between two groups were assessed by an unpaired *t*-test and among three or more groups by a one-way analysis of variance followed by Tukey’s Multiple Comparison Test. A *p*-value of less than 0.05 was considered as statistically significant. For the proteomics data, a Student’s T Test was used after logarithmic transformation.

## Data Availability

Supporting proteomic data can be obtained in the Supplementary results files.

## Supplementary Materials

The supporting information can be downloaded at: https://www.mdpi.com/article/10.3390/ijms232012126/s1.

## Abbreviations

AJ	adherence junctions
BBB	blood-brain barrier
BCA	bicinchoninic acid
BEC	brain endothelial cells
GTPases	small guanosine triphosphatases
iNOS and eNOS	inducible and endothelial nitric oxide synthase
LC-MS	liquid chromatography-mass spectrometry
LPS	lipopolysaccharide
NO	nitric oxide
TJ	tight junction
PBS	phosphate-buffered saline
PrPC	cellular human prion protein
ROS	reactive oxygen species
TEER	trans-endothelial electrical resistance
