# Supplementary material for: Cytoskeleton Elements Contribute to Prion Peptide-Induced Endothelial Barrier Breakdown in a Blood–Brain Barrier In Vitro System
Source: Int J Mol Sci. 2022 Oct 12;23(20):12126. doi: 10.3390/ijms232012126 (PMC9603506; doi:10.3390/ijms232012126)
Supplement: Supplementary file 1 [file ijms-23-12126-s001.zip › ijms-1921798-supplementary.pdf]

Table S1. Supporting Information: A list of the most significant (P-value < 0.05) proteins found to be differentially expressed in PrP-treated PBEC in comparison to untreated cells (n=3).

Protein accession number	Protein name and/or function	[1]Ratio PrP/control	T-test (P-value)
O62680	CD59- Cell lysis, apoptosis	1.56	0.0004
F1RTN8	Histone H4- Nucleosome-related	0.46	0.0067
F1SNF3	Podocalyxin-like protein 1(by homology to H) PODXL (gene name)- Regulation of adhesion and cytoskeleton	1.64	0.0069
Q2XVP4	Tubulin α1B	1.47	0.0124
F1SAG9	CD146(by homology to H) MCAM (gene name)- cell lysis, junction adhesion	1.66	0.0153
F1SDC7	Aldehyde dehydrogenase	1.62	0.0154
F1RK02	Plastin-2 (by homology to H)- actin-bindin protein)	1.83	0.0156
F1RI39	α-actinin-4 (by homology to H)- actin-binding protein	1.43	0.0184
P02543	Vimentin (intermediate filament)	0.58	0.0213
F1RN40	coagulation factor 7 isoform B protein (by homology to H) (proteolysis)	2.80	0.0225
B6VNT8	Cardiac muscle α actin 1	1.42	0.0234
P42639	Tropomyosin α -1 chain (actin- bindin protein)	1.41	0.0250
P18648	Apolipoprotein A-I	2.67	0.0257
F1SGJ5	Filamin B (actin-bindin protein)	1.46	0.0261
F1RFY1	Profilin (actin- bindin protein)	1.59	0.0307
P80272	Non-histone chromosomal protein HMG-17	0.59	0.0314
F1RS36	78 kDa glucose-regulated protein- protein traffic in the cell	1.35	0.0320
F1RX00	Amine oxidase [flavin-containing] A	1.63	0.0322
F1RWJ5	importin subunit beta-1 (by homology to H)-Trafficking: Mediates the nuclear import of HIV-1 Rev and Tat)	1.72	0.0337
F1S081	Integrin alpha-6 (by homology to H)- cell adhesion and signaling.	2.03	0.0343
A1XQU3	60s ribosomal protein L14	2.20	0.0352
Q29375	60s ribosomal protein L7a	2.12	0.0354
F2Z5D4	heterogenous nuclear ribonucleoprotein	1.16	0.0372
F1SQN1	T complex protein 1 subunit delta (folding of actin and tubulin)	1.54	0.0373
F1RX74	heterougeneous nuclear ribonucleoproteins A2/B1 (by homology to H)	1.24	0.0383
F2Z584	Histone H2B (Nucleosome-related)	0.21	0.0416

Q29549	Clusterin (cell lysis, apoptosis, Inhibits formation of amyloid fibrils by APP)	5.07	0.0424
F1RGA9	Coronin (actin-binding protein)	1.59	0.0441
F1RGM3	thioredoxin domain containing 17	1.97	0.0442
F1RMJ4	RAS-GTPase-activating-like protein IQGAP1(actin cytoskeleton reorganization, cell adhesion)	1.52	0.0447
F1S073	Anexin A2- Cytoskeleton-interacting protein, cell motility	1.79	0.0456
F1SAX3	Na/K-ATPase alpha subunit- transport, cell volume	1.85	0.0460
F1RTN3	Moesin (cactin-bindin protein, cell migration)	1.64	0.0483
F1RKM2	Lamin B1(intermediate filament)	1.55	0.0493

[1]Ratio of PrP/control was determined based on the average protein measurement in each group, which also passed the filtering criteria listed in Materials and Methods section. The uncharacterized proteins were run in BLAST and the top score was used for Name in the table.

Table S2. Supp.Info: Raw data of all hits in the LC-MS discovery experiment. PBEC were treated with 100µM PrP 106-126 (WT) for 24h. NT, control.

Protein Names	Protein MW	T-Test p value	Fold change (NT/WT)	Peptides Per Protein	Plgs Protein Score	Sequence Coverage [%]	Intensity CV in QCs [%]	Intensity [NT_01]	Intensity [NT_02]	Intensity [NT_03]	Intensity [WT_04]	Intensity [WT_05]	Intensity [WT_06]
CD59_PIG, SP:O62680	13790.2	0.000387	-1.56	2	12329	14.6	1.5	44411	45239	41159	70784	66856	65867
F1RTN8_PIG, TR:F1RTN8	11399.4	0.006737	2.46	4	23118	63.1	12.4	24652	15966	19691	8509	9806	9433
F1SNF3_PIG, TR:F1SNF3	46170.9	0.006923	-1.62	4	1506	22.7	9.1	30308	36419	36756	64063	48061	57505
TBA1B_PIG, SP:Q2XVP4	50151.6	0.012399	-1.61	14	32534	76.7	38.6	185179	208915	166313	281287	242560	301162
F1SAG9_PIG, TR:F1SAG9	71175.9	0.015346	-1.66	3	490	8.7	16.9	18018	23341	17500	33972	29591	34301
F1SDC7_PIG, TR:F1SDC7	53913.7	0.015385	-1.62	2	753	8.7	11.0	3321	4320	3274	6072	5080	6493
F1RK02_PIG, TR:F1RK02	70204.3	0.015636	-1.83	2	2708	3.5	16.6	525	738	550	1199	1003	1108
F1RI39_PIG, TR:F1RI39	87867.4	0.018423	-1.20	65	12653	81.6	3.0	1451548	1903231	1530505	2499727	2083491	2408530
VIME_PIG, SP:P02543	30989.4	0.021288	1.86	4	16756	25.5	6.2	17084	11436	14081	7257	9594	7895
F1RN40_PIG, TR:F1RN40	46980.2	0.022533	-2.80	2	629	4.3	40.1	1009	1948	1392	4080	3644	4464
B6VNT8_PIG, TR:B6VNT8	42019.0	0.023409	-1.46	11	53175	89.4	7.0	72444	89796	83403	125701	104449	119189
TPM1_PIG, SP:P42639	32706.6	0.02501	-1.41	2	3338	12.7	2.0	32918	39048	32354	56681	45343	45267
APOA1_PIG, SP:P18648	30325.4	0.025711	-3.12	2	717	4.9	12.8	nd peripher	15401	12828	38083	36675	38277
F1SGJ5_PIG, TR:F1SGJ5	186790.8	0.026127	-1.44	86	5420	69.5	2.5	1436973	1810907	1370337	2527237	1907249	2322340
F1RFY1_PIG, TR:F1RFY1	15043.3	0.030731	-1.61	18	36606	97.1	1.9	424317	594332	521107	926441	719668	799960
HMGN2_PIG, SP:P80272	9378.6	0.031429	1.69	2	18781	36.7	10.3	20271	15451	22431	10652	12308	11453
F1RS36_PIG, TR:F1RS36	70600.2	0.032017	-1.35	50	14348	64.4	1.7	1168179	1310067	1245435	2024441	1411659	1605785
F1RX00_PIG, TR:F1RX00	60030.2	0.032203	-1.63	3	574	10.4	6.6	18675	27256	24641	42251	36232	36595
F1RWJ5_PIG, TR:F1RWJ5	97225.2	0.033708	-1.72	3	471	4.8	10.4	14849	21783	18516	37408	24428	32864
F1S081_PIG, TR:F1S081	61067.5	0.034304	-2.03	3	1052	9.0	10.6	5710	8372	5064	17514	10607	10838
RL14_PIG, SP:A1XQU3	23329.9	0.035189	-2.20	2	764	18.8	8.6	6649	9427	8187	24556	13184	15547
RL7A_PIG, SP:Q29375	14580.1	0.035407	-2.15	4	1715	34.8	12.6	26611	37533	36725	99031	57293	57121
F2Z5D4_PIG, TR:F2Z5D4	62656.3	0.037201	1.27	10	1838	26.5	4.0	111223	126121	107257	142241	112143	143728
F1SQN1_PIG, TR:F1SQN1	58056.4	0.037277	-1.43	19	1891	48.4	3.5	161846	226784	183339	377500	227576	276396
F1RX74_PIG, TR:F1RX74	37519.8	0.038256	-1.45	8	5653	45.6	3.8	165395	185850	158524	233000	183848	214982
F2Z584_PIG, TR:F2Z584	13936.2	0.041617	4.85	2	23076	30.2	5.8	160778	63668	55780	7474	30989	19338
CLUS_PIG, SP:Q29549	51774.7	0.042381	-5.07	2	768	4.9	16.4	5129	4865	2153	37244	9370	15026
F1RGA9_PIG, TR:F1RGA9	53336.2	0.044074	-1.53	7	1010	17.3	7.3	81817	98364	86343	180599	116705	126637
F1RGM3_PIG, TR:F1RGM3	14078.1	0.044236	-2.10	4	4025	47.2	1.5	15500	24549	14753	48185	26444	33481
F1RMJ4_PIG, TR:F1RMJ4	77344.8	0.04467	-1.66	5	3609	13.6	2.6	53435	116968	61716	133617	107352	111717
F1S073_PIG, TR:F1S073	38880.3	0.045641	-2.63	19	16286	71.6	1.4	269978	513105	376072	877127	565578	629069
F1SAX3_PIG, TR:F1SAX3	112835.1	0.046021	-2.16	5	836	6.7	5.8	12271	12471	10609	31328	17981	16053
F1RTN3_PIG, TR:F1RTN3	67787.0	0.0483	-1.86	27	5500	47.1	11.4	499757	665096	527637	1275987	630678	873976
F1RKM2_PIG, TR:F1RKM2	66725.4	0.049267	-1.55	3	925	9.2	67.6	6379	5653	6107	11461	8649	7980
F1RQD0_PIG, TR:F1RQD0	13991.0	0.050023	-2.01	2	4232	31.1	7.0	8144	16518	10875	25644	18643	27230
UBC_PIG, SP:P0CG68	59993.0	0.052304	-1.57	9	180399	88.0	3.2	197998	298713	226817	380561	328022	412067
F1S4Y8_PIG, TR:F1S4Y8	37753.8	0.055285	-1.64	3	787	10.7	14.4	8130	8790	8872	12547	11934	17702
F2Z4Y1_PIG, TR:F2Z4Y1	28211.7	0.055576	-1.77	4	2698	22.8	6.1	71627	94013	66949	175184	101723	129318
F1SEX0_PIG, TR:F1SEX0	20545.6	0.056921	-1.50	3	1648	39.5	7.3	15324	17653	15156	29094	19664	23344
F2Z5T5_PIG, TR:F2Z5T5	50135.6	0.057339	-1.64	3	21794	53.0	6.0	14865	23514	21071	40048	25717	31957
F1RU19_PIG, TR:F1RU19	18417.4	0.058367	-1.49	2	52672	30.5	10.0	1790	2119	2136	2353	3367	3271
IBP3_PIG, SP:P16611	31690.2	0.058384	1.73	3	1518	20.8	7.7	61561	50114	42594	40498	24650	23932
VDAC2_PIG, SP:Q9MZ15	31592.5	0.058901	-1.76	4	2658	18.4	2.5	67632	111678	100436	174215	148111	146524
F1S8H8_PIG, TR:F1S8H8	32042.8	0.05911	-1.69	2	1899	12.2	5.5	16290	23824	17457	38751	23488	34946
RL31_PIG, SP:P62901	14462.9	0.059116	-1.27	4	3386	32.0	6.6	56244	64956	53453	79316	65725	77900
F1SFV1_PIG, TR:F1SFV1	43050.1	0.059228	-1.24	5	3306	16.9	6.3	60765	75301	67795	84772	75467	92761
CADH5_PIG, SP:O02840	87546.4	0.059501	-1.74	2	431	2.8	4.6	20937	26387	25392	55862	33284	37227
F1SDX9_PIG, TR:F1SDX9	21979.0	0.060491	-1.86	11	14719	84.9	4.0	171544	209295	175180	459682	251969	318279
F1SAA3_PIG, TR:F1SAA3	49606.1	0.062021	-1.81	10	2673	26.5	3.4	80698	98014	71613	186883	111699	137812
CATB_PIG, SP:A1E295	36900.6	0.064521	-1.36	5	2348	20.9	1.8	95484	135694	124474	173978	163779	162296
F2Z5B6_PIG, TR:F2Z5B6	32694.6	0.064884	2.22	5	3063	18.0	21.4	113056	105984	113434	41565	77656	37618
F1SUM7_PIG, TR:F1SUM7	21407.2	0.065226	-2.15	4	2950	41.8	3.2	10669	23156	14744	40684	27040	35323
F2Z5G5_PIG, TR:F2Z5G5	42613.7	0.065715	-1.49	3	1405	18.9	1.8	47703	72952	65741	97802	77891	101736
F1RUN2_PIG, TR:F1RUN2	69670.1	0.066277	-2.11	2	415	1.6	6.9	25988	35667	36143	100540	57305	48446
AK1A1_PIG, SP:P50578	36539.0	0.066984	-1.38	11	4659	53.5	5.0	152255	165721	159879	243597	191066	231603
F1S7E0_PIG, TR:F1S7E0	34537.1	0.067649	-2.95	2	1395	11.4	49.9	5143	3605	2148	13477	5083	13624
F1RIF8_PIG, TR:F1RIF8	53227.0	0.067735	-1.99	4	5026	11.2	1.4	61236	82335	39105	147331	92284	110280
C5H0C6_PIG, TR:C5H0C6	31284.1	0.067898	-1.41	2	4817	16.6	10.3	26615	35090	29772	46215	34413	47970
AT1A2_PIG, SP:D2WKD8	112208.4	0.068861	-1.63	2	620	3.7	11.8	3728	5865	4822	10014	6122	7313
F1SIT7_PIG, TR:F1SIT7	11528.0	0.069	-1.43	5	84854	81.6	11.9	62233	62755	71108	99773	73959	103632
F1RGK6_PIG, TR:F1RGK6	32864.9	0.070247	-1.90	3	6167	10.2	4.7	104705	110535	67403	204999	116483	215657
F1SUF2_PIG, TR:F1SUF2	102937.7	0.070348	-1.46	3	376	4.6	1.8	18339	27437	24695	32773	33103	37200
F1SDR7_PIG, TR:F1SDR7	28112.4	0.071234	-1.74	6	3495	30.5	6.4	118554	143540	133398	287626	169786	208157
F1S4R5_PIG, TR:F1S4R5	27379.5	0.071558	-1.65	2	6786	16.0	17.3	30551	46830	33809	61838	44988	76235
ECHA_PIG, SP:Q29554	83106.6	0.073191	-1.46	3	690	7.1	5.7	15020	23174	19376	30976	25583	27328
RL11_PIG, SP:Q29205	20212.3	0.073564	-1.86	3	3693	10.7	6.8	29736	33204	31347	80515	47141	48013
F1RK00_PIG, TR:F1RK00	180923.2	0.073777	1.59	9	3712	9.7	9.8	29081	25098	22292	20218	19527	21554
F1SFE6_PIG, TR:F1SFE6	45855.0	0.074595	-2.01	7	2130	20.8	12.3	16887	33890	18931	43332	36113	50040
C7AI81_PIG, TR:C7AI81	42009.0	0.076478	-1.98	7	43749	50.9	25.8	7139	12209	9282	23366	12334	16635
F1SJB5_PIG, TR:F1SJB5	38585.1	0.078419	-1.90	13	9392	55.8	5.5	85713	148598	123063	220409	157590	217144
F1S880_PIG, TR:F1S880	40570.8	0.07942	-1.48	2	1167	4.6	9.9	113800	180904	150803	260474	190005	208067
ENPL_PIG, SP:Q29092	92470.7	0.079648	-1.63	29	6090	40.8	1.2	277694	371777	340995	629093	385360	455705
MIF_PIG, SP:P80928	12451.4	0.080593	-1.22	4	19755	43.5	7.9	238772	249132	224840	328620	279289	263434
F2Z5K9_PIG, TR:F2Z5K9	15388.0	0.08063	1.75	2	22771	50.7	40.3	1199	872	836	503	387	772
THIO_PIG, SP:P82460	11828.6	0.080787	-1.67	4	5000	41.0	5.0	81660	107664	89234	201537	113936	150143
F1RX81_PIG, TR:F1RX81	85197.3	0.080787	-1.30	2	893	5.8	9.4	21302	26132	21932	35156	26326	28479
Q2VTP6_PIG, TR:Q2VTP6	11895.6	0.080985	-1.59	3	14458	13.0	16.4	39715	64607	57584	105594	66246	84890
F1RII0_PIG, TR:F1RII0	11064.4	0.083291	-3.97	6	26123	48.5	13.9	15000	49653	39753	265906	59671	86575
EIF1B_PIG, SP:P61220	12823.6	0.084259	-1.53	2	2227	37.2	7.4	11948	20016	15270	23518	22409	26373
F1S5J1_PIG, TR:F1S5J1	158700.1	0.085101	-1.97	2	524	3.9	7.6	1206	2159	1603	4755	2215	2834

F1SVB0_PIG, TR:F1SVB0	38942.1	0.085732	-1.59	3	958	14.9	7.2	14456	19860	13530	26862	18301	30678
B2CCY7_PIG, TR:B2CCY7	55180.8	0.086065	-1.72	2	1150	7.5	6.5	15797	19413	10709	34028	19330	25396
RL29_PIG, SP:Q95281	17497.7	0.08643	1.23	4	5084	26.2	5.3	34120	26700	31463	25570	24129	25383
F1SLA0_PIG, TR:F1SLA0	56335.6	0.087281	-1.39	36	10844	81.1	3.8	846634	1146358	970897	1460973	1104374	1262541
F1S2K3_PIG, TR:F1S2K3	14508.5	0.087548	-1.95	4	2425	42.5	7.0	11280	26086	17015	40203	32873	31670
F2Z558_PIG, TR:F2Z558	27745.1	0.088141	-1.44	17	9263	65.3	0.3	643795	910192	763711	1293564	866773	982217
F1S3E0_PIG, TR:F1S3E0	27129.2	0.088663	-1.52	4	5648	33.2	5.5	26145	41446	35647	64457	42121	48635
DX39B_PIG, SP:Q29024	48991.3	0.089133	-1.36	8	2987	27.1	7.0	80740	127882	90684	134499	122738	149943
HS71B_PIG, SP:Q6S4N2	70098.3	0.089846	-1.37	26	6854	57.9	3.4	499406	696345	643000	960313	733661	857711
F1S9S9_PIG, TR:F1S9S9	67705.5	0.090093	1.17	9	1762	15.8	5.3	80809	71901	75274	96896	67460	73055
RAB14_PIG, SP:Q52NJ6	23927.0	0.090418	-2.67	2	2611	9.8	3.3	4827	16055	8770	13499	32246	33481
F2Z5B1_PIG, TR:F2Z5B1	42570.9	0.096112	-1.24	3	467	8.5	4.2	24481	31960	30034	39367	34178	33582
F1SB42_PIG, TR:F1SB42	42268.8	0.096221	-2.51	2	814	6.5	5.4	6643	7431	5532	25954	9381	13800
F2Z5K6_PIG, TR:F2Z5K6	12253.7	0.097582	-2.05	2	1798	20.0	10.2	5013	9433	10006	25347	12930	11903
F1SPY4_PIG, TR:F1SPY4	51065.4	0.09826	-1.80	2	1113	6.1	5.5	6783	8607	7281	19368	10277	11070
F1RRD6_PIG, TR:F1RRD6	84501.9	0.098307	-1.50	2	564	3.7	5.0	10989	15115	12225	24573	14942	17861
F1S4X7_PIG, TR:F1S4X7	102888.2	0.098766	-1.35	2	8103	3.5	4.7	1938	2657	2145	2634	2797	3701
F1S827_PIG, TR:F1S827	42356.6	0.099414	2.29	18	6331	40.2	4.3	158463	288397	167317	156829	80473	156642
F1SA70_PIG, TR:F1SA70	69822.8	0.100308	-1.41	16	10857	32.2	9.7	617744	973763	747466	1229830	973585	1058987
RS12_PIG, SP:P46405	14514.9	0.101087	-1.75	6	11485	49.2	2.0	45933	69327	63851	151660	80234	79801
F1SDT8_PIG, TR:F1SDT8	39279.1	0.101089	1.23	3	733	10.5	10.8	34731	31625	27273	28832	23639	23923
F1SUE3_PIG, TR:F1SUE3	29113.0	0.101522	-1.80	7	4892	33.3	4.8	38168	63004	32851	95409	53318	83044
ALBU_PIG, SP:P08835	69692.2	0.10232	-1.59	4	2038	8.7	13.9	155725	144791	154487	349249	189594	215366
F1RI94_PIG, TR:F1RI94	15981.6	0.104261	-2.04	5	13751	51.1	2.4	32185	79477	73502	155604	99779	122067
F1RPX4_PIG, TR:F1RPX4	32523.9	0.104858	-1.40	9	2611	36.9	6.9	458574	549903	360371	719834	506120	703930
F2Z5E6_PIG, TR:F2Z5E6	22876.4	0.106381	-1.26	6	4297	28.4	3.0	82773	108373	86691	123824	100134	119866
F1SHD6_PIG, TR:F1SHD6	24804.8	0.106535	-1.27	7	5571	51.1	5.3	139401	150179	114156	179715	153348	190024
RL35_PIG, SP:Q29361	14549.5	0.107441	-1.52	2	10024	18.7	9.8	49979	80101	84405	115868	111616	98687
F2Z5I4_PIG, TR:F2Z5I4	14121.5	0.107731	-1.89	2	74117	23.8	9.2	84540	177508	196721	370958	223774	273940
F1S441_PIG, TR:F1S441	67596.5	0.108195	1.22	2	902	3.9	9.8	86345	107635	85649	72592	73546	83844
F1RYS5_PIG, TR:F1RYS5	49694.6	0.108673	-1.76	6	2473	13.0	4.1	17294	32985	30395	58304	37765	39712
F1RZR9_PIG, TR:F1RZR9	28312.3	0.108692	-1.37	8	3898	44.1	4.5	78253	111458	94762	157232	106581	142894
F1SFE3_PIG, TR:F1SFE3	57621.0	0.108711	-1.20	8	2074	21.2	11.6	53567	66049	55071	64055	66924	74343
F1STP1_PIG, TR:F1STP1	37320.4	0.109137	-1.35	3	1564	21.7	6.6	44218	53659	50429	79516	53509	67655
F1RRV6_PIG, TR:F1RRV6	34539.5	0.109145	-2.78	2	1490	17.8	18.7	10417	14287	5607	42683	12215	29483
F1SKF9_PIG, TR:F1SKF9	34531.5	0.109147	-2.02	2	2290	7.9	17.6	2777	5169	4974	13148	5536	7432
F1RSL7_PIG, TR:F1RSL7	82586.2	0.110533	-1.64	3	1583	3.6	8.6	10215	20804	13605	27681	18834	26658
F1S3U9_PIG, TR:F1S3U9	22395.7	0.111247	-1.91	10	5184	55.2	7.5	685450	921203	884651	2347855	1067608	1346482
F1RHW6_PIG, TR:F1RHW6	23192.8	0.112158	-1.54	3	3395	20.6	13.2	19781	26993	25250	49789	31116	29900
F1S8L9_PIG, TR:F1S8L9	88443.1	0.112728	-1.29	24	3927	35.6	1.3	616712	799370	686670	1036419	746165	801174
F2Z536_PIG, TR:F2Z536	10349.8	0.11286	-2.09	2	6284	37.1	6.1	17679	30391	29953	86668	37558	39053
F1RFQ7_PIG, TR:F1RFQ7	26066.9	0.114467	-1.22	6	2755	28.9	4.9	99832	121794	105432	145962	117091	133788
RSSA_PIG, SP:Q4GWZ2	32928.2	0.114902	-1.36	16	11459	58.3	6.3	249068	401997	291411	432670	384228	456383
F1RVE7_PIG, TR:F1RVE7	88243.1	0.114999	-1.67	3	1130	6.6	6.4	18331	24894	18817	47074	23909	32737
F1RTQ7_PIG, TR:F1RTQ7	14245.6	0.116925	-1.96	2	16220	46.6	24.6	1028	2765	1863	3934	3281	3844
F1RJU6_PIG, TR:F1RJU6	95466.1	0.11799	2.46	4	3218	5.2	9.5	9684	3530	6599	4617	2742	2591
F1SUM5_PIG, TR:F1SUM5	21364.2	0.121009	-2.01	3	3846	28.2	1.4	4612	10639	9545	23919	10401	15395
F2Z5J5_PIG, TR:F2Z5J5	12258.9	0.121344	-1.33	2	9070	24.0	3.8	14745	12960	14471	22217	15147	18532
RL23_PIG, SP:P62831	14865.4	0.122263	-2.53	5	4408	45.7	5.6	13110	64962	22108	50705	78162	87950
F1RP05_PIG, TR:F1RP05	45703.6	0.122342	-1.32	2	2077	14.8	16.7	20784	25906	17416	27001	27630	29721
F1SMZ6_PIG, TR:F1SMZ6	10959.7	0.123919	-1.95	3	5511	32.4	8.8	27429	56876	44240	128684	64477	57634
A6M928_PIG, TR:A6M928	46153.9	0.124355	-1.21	16	5618	55.2	3.5	254183	355709	289806	431425	338571	377217
TCTP_PIG, SP:P61288	19595.3	0.12451	-3.13	9	23595	60.5	21.0	34631	80732	46556	190154	84212	128047
F2Z554_PIG, TR:F2Z554	12784.1	0.124961	-1.62	9	20843	67.0	5.3	46620	95102	74121	99640	105845	122475
F1S3B4_PIG, TR:F1S3B4	40206.9	0.125266	-1.67	3	1692	19.8	17.2	3377	6865	3889	7778	5723	10130
F1RUX8_PIG, TR:F1RUX8	37670.2	0.125813	-1.40	3	1912	16.9	3.8	35734	42681	37543	69752	46858	46194
2AAA_PIG, SP:P54612	65322.6	0.126634	-1.23	8	1145	18.8	2.4	112353	136319	103802	167481	124813	139787
F1RYZ7_PIG, TR:F1RYZ7	18401.6	0.126819	-1.70	2	7494	28.6	6.6	946	1663	1737	3384	1759	2226
S10AB_PIG, SP:P31950	11179.8	0.126915	-2.83	2	5652	15.2	17.0	7799	16591	15916	69456	18979	25696
HSP76_PIG, SP:Q04967	71109.2	0.128758	2.88	17	7384	39.3	4.7	104956	66355	45562	33539	50283	55102
RS18_PIG, SP:P62272	17718.7	0.129217	-2.09	2	1492	13.8	32.1	7056	20492	18618	45164	25060	26077
F1RHC5_PIG, TR:F1RHC5	39675.1	0.129677	-1.46	6	8901	22.0	3.2	75864	83203	47812	124330	79841	82919
B5APU6_PIG, TR:B5APU6	40974.7	0.131173	-1.62	2	671	8.3	12.9	27022	41097	40503	82563	49042	44268
F1SSX0_PIG, TR:F1SSX0	37234.7	0.131603	-1.18	3	1555	17.7	4.8	26575	27578	22632	31881	26521	32363
F1SU07_PIG, TR:F1SU07	7028.0	0.132302	-1.50	2	6399	30.6	9.1	9007	16113	9431	18812	13965	19048
F1RFP0_PIG, TR:F1RFP0	20803.9	0.133032	-1.90	2	18955	37.0	27.2	765	2121	1268	2593	3506	1793
F1S9D6_PIG, TR:F1S9D6	223135.2	0.133947	-1.46	2	8107	1.7	4.4	13358	11055	7414	16239	13790	16494
F1SBW4_PIG, TR:F1SBW4	33341.7	0.13405	-1.39	2	1864	9.9	3.8	22941	34094	22115	44825	30517	34894
F1RQG6_PIG, TR:F1RQG6	37540.1	0.134348	-1.46	18	11292	65.4	11.6	611254	1024708	950250	1200261	1019706	1392403
F1ST98_PIG, TR:F1ST98	19934.4	0.134888	-1.40	2	1884	14.9	4.0	7290	11994	10841	15191	13451	13600
F1RPG9_PIG, TR:F1RPG9	44765.8	0.135539	-1.62	14	24855	70.4	11.1	127616	204530	176630	257252	214210	243784
RL6_PIG, SP:Q2YGT9	32190.2	0.135554	-1.35	3	1950	12.0	6.9	26206	38503	25468	47865	35928	37833
F2Z522_PIG, TR:F2Z522	17695.1	0.137594	-2.41	4	12159	21.2	5.3	39268	32585	15044	94241	44358	36866
F2Z5Q6_PIG, TR:F2Z5Q6	28680.6	0.138421	-2.25	3	892	15.3	1.1	21377	20835	15240	72567	26721	30261
RS28_PIG, SP:Q6QAT1	7841.0	0.138951	-1.96	7	7415	71.0	0.6	37719	52083	43523	140949	54078	66318
F1SK86_PIG, TR:F1SK86	51289.0	0.13908	-1.51	5	933	18.9	7.0	33604	33148	32324	70182	36744	41639
LDHA_PIG, SP:P00339	36618.6	0.139292	-1.42	3	4099	18.7	8.9	13678	20865	16884	23039	19043	30916
F1SNL7_PIG, TR:F1SNL7	52734.3	0.139867	-1.75	4	741	11.4	5.0	17276	24023	17570	59884	23196	30729
F1RY92_PIG, TR:F1RY92	19230.5	0.139947	-1.48	6	11117	40.2	12.3	91553	115804	75428	143604	99750	173316
LDHB_PIG, SP:P00336	36612.4	0.1401	-1.38	12	7422	54.2	3.9	302854	495475	415274	674563	520086	626947
F1RP93_PIG, TR:F1RP93	27930.7	0.141573	-1.74	2	867	11.4	2.6	2204	3956	3031	7838	3663	4491
D0G0C8_PIG, TR:D0G0C8	57444.2	0.141729	-1.61	7	1222	23.7	6.7	94696	116771	110140	255189	119911	166348
F2Z571_PIG, TR:F2Z571	49831.0	0.14238	-1.20	17	35388	78.2	4.5	497936	616720	543775	751210	598727	610839
F1SJ34_PIG, TR:F1SJ34	16748.2	0.14257	-1.28	4	4274	31.6	10.1	73127	100099	96327	115040	122537	106557
MOES_PIG, SP:P26042	67660.8	0.143232	-1.51	6	2669	18.9	1.4	207594	217679	182162	426086	237015	313444
F1SHL9_PIG, TR:F1SHL9	11878.5	0.143269	1.56	2	6577	39.8	5.0	717979	432484	768386	524381	401983	301253

CAN2_PIG, SP:P43367	37808.8	0.143393	-1.59	2	2601	16.4	2.5	14389	23215	12413	30326	18067	31038
F1RGC0_PIG, TR:F1RGC0	16605.5	0.144334	-1.38	3	3014	17.6	4.3	33955	53442	51585	77574	60633	54164
A5D9J7_PIG, TR:A5D9J7	80212.1	0.144739	-1.57	2	563	6.2	6.0	9177	10604	7329	18456	9528	14692
PRS8_PIG, SP:P62197	45626.0	0.145158	-1.39	3	686	12.8	5.1	39509	44029	36005	70701	44029	50920
F1SKM0_PIG, TR:F1SKM0	52698.5	0.145318	-1.43	2	1578	6.5	6.6	23984	29012	16328	32151	31773	35368
F1SSZ7_PIG, TR:F1SSZ7	94829.5	0.145785	-1.25	4	1211	5.9	4.9	69003	63242	60733	99532	74000	78986
F1RWC5_PIG, TR:F1RWC5	109252.3	0.148747	-1.26	7	1061	9.6	11.7	24930	33518	24371	49715	36303	30120
F2Z5H9_PIG, TR:F2Z5H9	49229.5	0.14879	-1.56	14	7113	55.2	3.0	107187	108919	104700	224987	114044	131163
MYL6_PIG, SP:P60662	16930.1	0.149099	-1.41	15	20836	88.1	12.7	836269	993031	977688	1461345	1013737	1290577
F1SBA5_PIG, TR:F1SBA5	27928.0	0.149237	-1.71	2	2733	10.0	7.7	7508	12285	11148	26190	13533	13064
RLA0_PIG, SP:Q29214	34358.6	0.149923	-1.98	2	1480	10.7	3.5	3069	8932	8727	18118	11392	11458
F1RQU2_PIG, TR:F1RQU2	83253.2	0.14997	-1.32	35	8205	57.7	7.4	532247	865120	730736	1131295	806846	1001282
F1S395_PIG, TR:F1S395	30954.7	0.150816	-1.36	4	2651	15.0	4.0	69166	86793	83198	136278	84719	110691
AT1A1_PIG, SP:P05024	112680.9	0.151807	-1.28	6	819	8.5	6.8	51366	61495	52969	81737	62902	82297
F1RZQ0_PIG, TR:F1RZQ0	134871.2	0.153344	1.32	23	9723	34.0	4.9	441886	573693	458984	556079	465466	445285
F1SIH8_PIG, TR:F1SIH8	63582.1	0.153729	-1.51	3	8820	15.1	8.3	42827	87172	64081	99891	89446	103970
F1RPJ5_PIG, TR:F1RPJ5	21858.4	0.154198	-1.60	2	2202	26.6	20.7	12791	22749	15537	38115	19503	24104
F1S4K7_PIG, TR:F1S4K7	69859.0	0.155907	-1.39	8	3221	23.6	2.6	125134	207983	143552	218744	185737	261438
F1RW72_PIG, TR:F1RW72	38368.5	0.155931	-1.50	6	2179	20.5	5.4	58255	58615	61925	108311	60051	86073
F1S529_PIG, TR:F1S529	35426.9	0.156337	-1.62	3	6620	30.7	2.4	29226	61026	46465	99939	53862	67466
F1RFX8_PIG, TR:F1RFX8	29078.7	0.157049	1.33	8	11397	36.3	17.5	154121	157048	123355	173092	80885	179495
F1SMN0_PIG, TR:F1SMN0	37425.2	0.15731	-1.33	8	10732	32.1	24.4	57671	73645	70248	94955	54197	72797
F1SLY7_PIG, TR:F1SLY7	20665.2	0.159276	-1.33	2	1741	17.6	2.7	8792	10628	9562	15182	9848	13447
OAS1_PIG, SP:Q29599	40246.4	0.159398	1.37	2	3035	14.0	2.7	31947	41822	33145	18646	30473	29071
F1RIP4_PIG, TR:F1RIP4	48719.8	0.161972	-1.66	4	751	11.8	2.3	13512	22002	14239	19120	22130	25864
COF2_PIG, SP:Q5G6V9	18736.6	0.162474	-1.27	6	9669	36.1	4.4	28126	36358	31817	45700	33533	38776
F1SQS2_PIG, TR:F1SQS2	50679.3	0.163939	-2.24	6	3266	22.2	2.7	28316	28768	16438	72272	23149	52723
CO1A2_RAT, SP:P02466	129564.1	0.164557	1.56	2	503	2.3	2.8	54951	32200	52848	42204	26745	20837
F1RWW4_PIG, TR:F1RWW4	63564.6	0.165355	-1.46	4	805	11.2	7.2	37814	55058	43381	93649	50781	60804
RAB1A_PIG, SP:Q52NJ2	22605.7	0.165993	-1.89	3	3178	25.4	12.4	8763	30528	18280	38575	28746	41726
H33_PIG, SP:Q71LE2	15327.9	0.167902	3.01	3	35278	64.0	18.5	284303	152016	111815	11698	77708	92785
G3P_PIG, SP:P00355	35836.0	0.168846	-1.51	37	59327	86.8	8.3	2232472	3250654	2839632	4567495	3132601	3346532
F1SMN5_PIG, TR:F1SMN5	290412.4	0.169203	-2.19	2	653	0.7	54.0	5594	29979	10792	22785	38856	40126
F1SS26_PIG, TR:F1SS26	130311.6	0.170805	-1.21	81	8227	70.5	5.5	2449447	2830330	2438597	3667539	2535645	2856344
F1RQZ9_PIG, TR:F1RQZ9	109591.0	0.171289	-2.06	3	663	5.7	6.9	21394	29224	16228	77684	31134	28842
F1RUZ2_PIG, TR:F1RUZ2	54151.5	0.172579	-1.17	3	1351	17.4	5.9	45522	39735	43324	55702	43194	51096
HSPB1_PIG, SP:Q5S1U1	22941.8	0.173498	-1.19	11	31293	94.2	3.8	1379676	1829965	1551034	2260529	1739091	1830800
F1S1X9_PIG, TR:F1S1X9	32281.4	0.173834	-1.28	9	5531	29.4	2.4	100672	144593	104289	162072	121146	183404
CPNS1_PIG, SP:P04574	28068.5	0.174809	-1.87	2	934	20.3	25.4	6172	10947	9658	24953	9268	15962
F1RKI3_PIG, TR:F1RKI3	13724.8	0.175226	-1.22	8	10263	81.7	2.2	239598	326789	280546	347960	310217	363745
D3K5N3_PIG, TR:D3K5N3	15597.2	0.176881	-1.65	2	4091	21.0	3.7	7847	8931	8060	20149	9950	10855
F1SS61_PIG, TR:F1SS61	223848.8	0.17718	-1.46	6	8105	5.4	2.7	5803	7742	6218	13481	7686	8378
DHSA_PIG, SP:Q0QF01	72831.6	0.178618	-1.17	2	1075	7.4	6.3	9402	12142	11563	14399	11755	12699
F1SDX6_PIG, TR:F1SDX6	77224.3	0.17878	-1.44	28	4088	45.6	2.8	349746	441987	335008	653884	416081	618259
RS16_PIG, SP:Q29201	16445.3	0.180597	-1.96	3	1228	20.5	9.1	10072	36139	31322	65110	38717	48382
NB5R3_PIG, SP:P83686	30830.7	0.180918	-1.38	6	16372	40.1	15.2	45262	68440	55651	92916	57360	76752
F1S5R2_PIG, TR:F1S5R2	13938.1	0.181087	-2.18	2	28100	58.7	10.1	1473	8554	2408	5773	9683	11618
F1SHL8_PIG, TR:F1SHL8	62798.2	0.181681	-1.46	13	34937	52.9	3.0	169618	222434	194718	364113	215476	236488
UCHL1_PIG, SP:Q6SEG5	24859.3	0.182187	-1.72	8	8094	37.2	4.0	48128	65775	65291	127294	69621	82327
F1SAP6_PIG, TR:F1SAP6	53489.9	0.18229	-1.41	3	4606	11.6	5.8	13847	22931	12496	24765	18515	26045
F2Z545_PIG, TR:F2Z545	12473.2	0.182318	-1.36	4	3809	50.0	8.0	17154	29027	25989	38317	30205	34941
RS21_PIG, SP:P63221	9111.4	0.184014	-1.28	2	6365	22.9	6.7	37393	42554	31893	59680	41363	42127
F1SEV8_PIG, TR:F1SEV8	31032.7	0.186063	-1.19	12	9887	44.3	3.7	229591	271799	226505	338016	251415	261719
F1SN37_PIG, TR:F1SN37	88125.9	0.186714	-1.64	3	728	5.7	10.6	10686	14984	12844	31638	14888	16739
F1RWY8_PIG, TR:F1RWY8	47999.7	0.186881	-1.62	2	19810	32.3	15.6	3529	8845	7638	10842	8876	12740
F2Z5J1_PIG, TR:F2Z5J1	49184.5	0.187908	-1.51	3	1411	7.7	8.9	16436	38047	23134	42832	31914	42635
F1S9A4_PIG, TR:F1S9A4	50022.3	0.188881	-1.70	2	731	8.6	9.4	5662	14909	14656	20605	21737	17704
F1RZ71_PIG, TR:F1RZ71	23134.1	0.189437	-1.72	2	748	14.5	4.3	3643	7342	3390	10648	4776	9365
F1SGD7_PIG, TR:F1SGD7	45374.2	0.190468	-1.66	9	3517	30.7	7.6	44578	99045	55664	91822	87271	118617
F1RTJ9_PIG, TR:F1RTJ9	18911.3	0.191834	-1.34	5	11735	34.4	4.9	65779	112279	93620	143054	119744	109267
E1CAJ5_PIG, TR:E1CAJ5	56858.7	0.192372	-1.28	41	7029	71.9	3.2	941151	1148207	966007	1672296	1083924	1244560
F1RL32_PIG, TR:F1RL32	11547.4	0.196515	1.50	2	1052	11.4	5.3	2813	2356	2293	2411	1024	1535
Q06AS6_PIG, TR:Q06AS6	40464.9	0.197643	1.28	23	12278	66.2	1.0	345881	372778	308012	369943	375560	401361
F1RK08_PIG, TR:F1RK08	31681.0	0.197762	-1.36	3	3520	25.7	16.9	16545	23140	15732	24800	18857	31802
F1RQP0_PIG, TR:F1RQP0	21234.6	0.198594	-1.25	3	2172	30.8	7.2	9177	13844	9956	12312	13339	15510
CAV1_PIG, SP:Q6RVA9	20624.8	0.19873	-1.63	2	2558	24.2	7.1	62559	76252	74570	172843	81184	92751
F1RWI5_PIG, TR:F1RWI5	29410.1	0.199176	-1.36	3	4078	12.4	3.7	61184	115433	77128	100566	119142	126592
F1SRC5_PIG, TR:F1SRC5	85511.5	0.201238	-1.25	7	1774	12.7	1.7	56270	62214	58613	82661	56223	65775
RADI_PIG, SP:P26044	68549.8	0.201302	-5.64	2	2027	5.5	25.1	525	853	622	8862	955	1463
F1RZ28_PIG, TR:F1RZ28	18931.8	0.202365	-1.50	2	825	9.7	12.3	8214	17765	17000	23807	19212	21547
H4_PIG, SP:P62802	11367.3	0.205005	-1.37	7	35102	67.0	8.6	1106987	2058984	1809739	2738747	2369419	1899385
CAZA2_PIG, SP:Q29221	32981.1	0.205639	-1.42	4	2714	25.2	8.2	55343	68774	59512	115070	64507	81698
F1SBY5_PIG, TR:F1SBY5	49854.7	0.206168	-1.52	2	1143	8.9	29.9	6663	7567	4197	7182	7471	13419
HS90A_PIG, SP:O02705	84774.8	0.207106	-1.29	45	10428	66.2	8.3	455240	845313	705220	1105315	921943	1059035
ACTS_PIG, SP:P68137	42051.0	0.207901	-2.01	13	97441	76.9	10.8	295841	351114	336980	895738	366604	432932
F1RI15_PIG, TR:F1RI15	94496.3	0.208352	-1.18	11	1878	24.9	4.5	96216	102409	92209	106154	95415	127410
F1RWE1_PIG, TR:F1RWE1	85237.0	0.208772	-1.31	3	476	7.7	7.8	13596	16712	13760	23188	14384	19999
F1SML4_PIG, TR:F1SML4	66086.6	0.209766	-1.21	5	1839	13.3	2.7	32139	42004	35099	39730	31628	49690
F2Z5J9_PIG, TR:F2Z5J9	24488.5	0.209833	-2.39	2	2214	10.1	9.9	7157	10922	16007	51387	14448	15579
F1SB53_PIG, TR:F1SB53	53065.9	0.210492	-2.29	2	1116	6.3	4.6	7079	8337	3338	25082	6319	11557
F1RYZ0_PIG, TR:F1RYZ0	11693.0	0.210734	-1.52	3	7444	40.0	13.0	16022	39405	30220	51620	42226	36278
F1SMC0_PIG, TR:F1SMC0	40964.0	0.211151	-1.48	12	10626	51.6	5.2	99735	161839	137735	223815	155027	178134
F1S9Q3_PIG, TR:F1S9Q3	68494.1	0.211934	-1.41	50	24157	69.6	1.4	1157920	1737859	1402363	2840991	1751023	2161470
F1RL04_PIG, TR:F1RL04	47632.5	0.212013	-1.61	2	1623	6.4	4.1	13399	33109	35071	45165	40221	46049
F1SR80_PIG, TR:F1SR80	50337.9	0.212597	2.09	10	21317	34.3	1.0	63403	31078	33873	34006	20562	26316
F1SAD9_PIG, TR:F1SAD9	72909.4	0.213665	-1.64	7	974	11.0	10.4	59959	72680	68867	163124	74140	101774

F6Q5P0_PIG, TR:F6Q5P0	17222.3	0.215085	-1.65	3	2930	21.9	6.5	9385	25229	26127	31453	35476	33460
F1RMT1_PIG, TR:F1RMT1	42549.5	0.216008	-1.26	2	639	12.1	14.1	27709	29067	25578	43760	29833	30398
F2Z550_PIG, TR:F2Z550	24831.3	0.21744	-1.63	2	6810	23.5	7.2	14305	24813	34538	58999	30640	30191
RTCB_PIG, SP:Q19PY3	55238.3	0.219354	-1.86	3	2536	17.6	5.8	18290	18480	15768	52663	19007	25971
SODC_PIG, SP:P04178	15891.7	0.220007	-1.36	14	37344	87.6	5.6	226485	352881	305440	553984	329684	354763
F1SQK1_PIG, TR:F1SQK1	123004.0	0.221916	-1.83	2	1239	7.6	4.9	234	900	609	1551	849	783
F1SLU6_PIG, TR:F1SLU6	78003.1	0.222329	-1.23	2	621	7.2	14.6	10580	14736	9514	13817	13845	15325
A6M930_PIG, TR:A6M930	46402.3	0.222534	1.21	12	5368	48.6	3.0	230031	278844	233687	172510	222939	222905
RL10_PIG, SP:Q29195	24602.9	0.223727	-1.36	11	16648	50.0	2.7	127335	197368	127900	247647	156434	214094
GBLP_PIG, SP:P63246	35076.7	0.224092	-1.31	11	2408	51.7	2.6	89807	149932	135836	193504	166610	181805
GDIB_PIG, SP:Q6Q7J2	50267.8	0.224185	-1.29	8	1834	32.6	5.9	170239	225802	223364	292357	217009	232101
F1RU33_PIG, TR:F1RU33	10044.5	0.224386	-1.89	6	11093	59.6	7.9	51025	60493	45954	158758	56116	69989
F2Z5G3_PIG, TR:F2Z5G3	16837.6	0.227454	-1.66	13	48663	94.6	23.8	166664	298794	170260	239827	233188	467002
F1RSH7_PIG, TR:F1RSH7	30092.0	0.228601	-2.15	8	22528	38.1	25.2	38121	123042	51341	70613	135618	162167
F1S340_PIG, TR:F1S340	21733.4	0.229705	-1.91	2	1001	16.1	8.2	3396	2975	4789	11629	5927	3719
F1S9C3_PIG, TR:F1S9C3	37226.3	0.2301	-1.61	6	3340	32.2	3.0	39053	114837	94396	135044	150075	129785
F1RVN0_PIG, TR:F1RVN0	23436.9	0.230373	-1.31	6	12605	56.9	4.0	140311	251697	224219	299648	242169	278983
F1RQZ0_PIG, TR:F1RQZ0	62952.1	0.231832	-1.90	2	1536	7.5	2.6	30420	40666	28725	106936	36232	46952
Q8WNW4_PIG, TR:Q8WNW4	85510.6	0.232472	-1.41	6	1302	14.5	8.7	76254	74470	65600	139491	68905	81491
F2Z5G8_PIG, TR:F2Z5G8	13742.1	0.233249	-1.80	2	2939	15.2	6.6	1146	1349	1279	3715	1643	1440
F1SEN2_PIG, TR:F1SEN2	61307.8	0.233255	-1.14	21	5958	49.1	3.1	401271	504223	425311	580848	473993	554535
F1S956_PIG, TR:F1S956	27534.9	0.234078	-1.97	4	2272	24.8	3.8	13574	14400	11620	43491	13831	21885
F1RJX8_PIG, TR:F1RJX8	138440.9	0.235968	-1.24	5	1386	7.8	7.4	31474	46391	29953	46858	42326	50550
HS71L_PIG, SP:A5A8V7	70343.9	0.236222	-1.22	3	1600	15.4	10.0	8607	13483	10696	15004	12216	12885
F1RIS1_PIG, TR:F1RIS1	52594.1	0.236817	-3.00	2	2441	7.3	14.4	16458	28327	18153	132190	28476	28250
F1RR78_PIG, TR:F1RR78	284350.0	0.236837	-1.58	36	1252	23.5	7.6	453045	525026	421013	889552	510026	701337
GELS_PIG, SP:P20305	84775.4	0.236915	-1.18	11	3870	26.2	1.7	45664	66847	59492	108491	66227	73699
F1STN7_PIG, TR:F1STN7	50266.1	0.237646	-1.59	4	3918	13.2	5.5	220003	248497	274960	602746	282763	296292
F1SNY2_PIG, TR:F1SNY2	46392.5	0.238604	-1.21	6	2116	14.8	4.9	72593	107117	80273	113963	98022	103738
F2Z4Y0_PIG, TR:F2Z4Y0	13916.2	0.238923	-1.45	2	4911	10.3	14.5	22178	14779	13223	33964	17287	21373
PECA1_PIG, SP:Q95242	82378.8	0.239333	-1.39	7	1664	12.4	2.7	126154	146103	111058	248730	134557	154083
RS26_PIG, SP:P49171	13015.4	0.240475	-2.09	3	5120	27.0	29.5	1761	13736	6946	9004	19906	17908
F1SFA7_PIG, TR:F1SFA7	129159.6	0.241311	1.66	3	732	2.6	5.6	154979	66146	150713	88999	77453	57192
CISY_PIG, SP:P00889	51629.5	0.245191	-1.39	4	4457	10.1	5.3	25862	26436	27252	50776	28170	31208
MDHM_PIG, SP:P00346	35603.2	0.245404	-1.35	24	11846	70.4	5.2	203710	275954	248524	428594	276511	311309
F1S6M7_PIG, TR:F1S6M7	50418.7	0.245543	1.26	14	10270	51.8	3.6	65359	78801	94741	79789	68998	72157
CDC42_PIG, SP:Q007T2	21258.6	0.250457	-1.40	4	1859	27.2	23.1	67702	49985	53383	117081	62619	62877
COF1_PIG, SP:P10668	18518.6	0.250994	-1.40	9	49561	83.1	6.8	111982	125074	112592	209201	117306	143152
F1S0A3_PIG, TR:F1S0A3	54041.2	0.252701	-1.29	2	713	5.8	6.6	4462	7179	8005	9386	7813	8147
F1RQR4_PIG, TR:F1RQR4	62771.9	0.255201	-1.61	6	1528	20.8	21.0	14444	40846	17951	25058	32239	44302
RL15_PIG, SP:P79324	17837.3	0.256142	1.29	2	2965	16.3	14.4	35210	44471	33274	20436	37852	29036
F1SJR5_PIG, TR:F1SJR5	55588.6	0.257338	-1.67	7	1877	20.0	6.7	27595	30206	27496	59296	32682	41444
F1S5Z3_PIG, TR:F1S5Z3	61197.1	0.257743	-1.44	3	880	8.6	4.7	16072	20608	14061	31830	15509	25800
F1S7W0_PIG, TR:F1S7W0	186359.3	0.258282	-1.30	21	1430	27.4	12.0	74771	120455	56518	122914	79972	133650
DDC_PIG, SP:P80041	53936.3	0.258401	-1.23	3	2002	7.0	4.2	17581	18719	14550	25448	16749	20105
F1RWC0_PIG, TR:F1RWC0	53667.7	0.25898	-1.29	70	36084	96.8	5.7	12880000	12912000	9474491	18631000	11642000	14690000
F1SKI0_PIG, TR:F1SKI0	226792.8	0.25969	-1.48	110	13715	59.8	7.0	1777852	2306670	1807888	4089270	1713372	3021128
F1SSH7_PIG, TR:F1SSH7	27559.7	0.262774	-1.61	2	2810	19.9	27.1	2075	8518	3394	5713	7853	8903
F1S8H5_PIG, TR:F1S8H5	35301.2	0.263871	-1.72	2	1295	9.1	15.3	4265	19921	11729	15269	18331	28179
F1SAT8_PIG, TR:F1SAT8	70295.9	0.264177	-1.48	3	472	8.0	6.3	11933	14106	11549	26596	12173	16900
F1SM03_PIG, TR:F1SM03	34450.1	0.267037	1.75	2	865	12.5	8.6	19868	6873	13154	8148	7319	7361
F1S895_PIG, TR:F1S895	64882.7	0.268451	-1.60	3	718	9.7	14.9	7204	9492	6653	17024	6816	13603
F2Z5P1_PIG, TR:F2Z5P1	13377.5	0.268664	1.53	2	13006	20.5	6.0	41279	21530	18600	14689	18793	19827
CAPZB_PIG, SP:A0PFK7	31315.4	0.268798	-2.31	5	8538	23.5	6.9	39307	48856	5483	111243	55627	46132
F1SNK3_PIG, TR:F1SNK3	31238.2	0.270186	-1.40	4	2431	21.2	9.0	35535	35753	30688	64997	32531	45277
F1RST0_PIG, TR:F1RST0	96698.5	0.273329	-1.22	4	1060	7.6	2.0	47889	59050	37633	67193	51708	57042
F1SEG5_PIG, TR:F1SEG5	16559.4	0.274543	-1.37	2	1586	20.5	17.7	39022	58254	42888	43933	63099	85351
F1SLF0_PIG, TR:F1SLF0	28774.2	0.276494	-1.33	2	2181	9.4	7.3	15921	17656	24337	33165	18419	25243
F1SPF9_PIG, TR:F1SPF9	52583.8	0.277502	-1.07	4	1879	14.0	5.2	44067	48593	46778	54416	44705	52755
F1S3L0_PIG, TR:F1S3L0	100185.5	0.278345	1.80	3	5055	6.2	13.6	108066	115629	102631	14983	94128	72124
F1SS67_PIG, TR:F1SS67	223547.6	0.279091	-1.53	3	8141	2.2	12.5	10248	12802	7899	15010	9459	23008
TBB5_PIG, SP:Q767L7	49670.8	0.279391	-1.17	18	30095	71.4	8.7	718768	880681	706937	1185147	765430	1016164
F2Z5K4_PIG, TR:F2Z5K4	22006.4	0.280913	-1.29	7	5413	26.4	5.0	78545	146329	114431	155792	152177	128182
F1S9S5_PIG, TR:F1S9S5	58877.0	0.281329	-1.56	3	1277	7.2	6.1	13094	18271	15944	38582	16465	18942
F1RFN9_PIG, TR:F1RFN9	54542.0	0.281571	-1.14	33	7007	74.8	5.5	635670	829880	678681	1023705	771390	894470
AATM_PIG, SP:P00506	47436.4	0.282314	-1.24	9	3188	40.5	2.4	158621	204826	180664	290495	195421	195944
F1SGJ3_PIG, TR:F1SGJ3	26138.5	0.282402	-1.12	16	14252	71.6	3.6	310563	345321	269040	412810	348205	379947
F1RRK9_PIG, TR:F1RRK9	136287.9	0.283161	-1.30	6	1524	7.0	4.6	22974	41275	29225	43295	35321	44725
F1SS64_PIG, TR:F1SS64	223164.3	0.286029	1.14	5	8147	3.9	4.9	13472	17112	13778	11754	14820	15142
ANXA1_PIG, SP:P19619	38759.2	0.286765	-1.54	13	12516	43.6	3.7	226726	240795	165378	407228	179894	301683
F1SMB9_PIG, TR:F1SMB9	20250.9	0.289309	-1.36	8	5194	67.7	17.4	51084	57891	34319	65677	43092	76768
Q2XVP5_PIG, TR:Q2XVP5	29977.1	0.289569	1.24	7	7443	39.6	5.3	158276	151995	117843	161605	126630	104335
PGK1_PIG, SP:Q7SIB7	44558.6	0.290501	-1.40	11	26063	58.5	3.2	76751	121908	108589	205187	119478	125579
F2Z5B4_PIG, TR:F2Z5B4	39652.0	0.291676	-1.64	2	1668	7.9	20.4	11849	48012	32553	72790	36650	41742
RL18_PIG, SP:Q95342	12242.1	0.292692	-1.51	4	5631	39.4	19.3	24810	65281	29234	37697	61609	80151
B5APU3_PIG, TR:B5APU3	44760.8	0.292718	-1.23	7	1820	28.7	6.4	100199	113532	109622	184800	130770	115944
F1SKJ1_PIG, TR:F1SKJ1	226489.2	0.292955	1.20	31	11978	42.7	4.7	334379	328072	324369	258149	284631	348548
F1SSA6_PIG, TR:F1SSA6	216124.5	0.293386	1.19	36	8679	22.9	7.3	268263	294210	264204	323031	218284	280130
F1SG38_PIG, TR:F1SG38	53143.9	0.293836	-1.23	6	2581	19.8	8.6	17624	26040	26854	33537	23517	28446
F1SPG2_PIG, TR:F1SPG2	68625.6	0.294294	-1.45	4	1020	10.0	9.5	47533	38473	37366	85659	44014	48268
TPIS_PIG, SP:Q29371	26729.6	0.295644	-1.74	16	7805	81.0	1.3	79249	95637	83515	257455	104637	108830
F1RHI6_PIG, TR:F1RHI6	56889.7	0.295796	-1.84	8	1774	33.5	10.3	29059	46870	7558	53572	46633	63161
F1S0N1_PIG, TR:F1S0N1	119817.2	0.300248	1.36	3	587	3.7	13.8	24141	17318	22383	24821	11666	10547
F1S3W0_PIG, TR:F1S3W0	10670.8	0.300451	-1.18	3	9284	48.4	6.6	28394	30588	21526	33865	26117	35083
F1S3J9_PIG, TR:F1S3J9	58434.5	0.304353	-1.20	3	1577	16.6	7.1	42455	41208	34130	57968	37934	45533
Q9N0Y9_PIG, TR:Q9N0Y9	39639.8	0.309267	-1.24	11	6806	40.1	2.0	71552	94022	68921	133733	79925	102338

PP2AA_PIG, SP:P67776	35594.2	0.310647	-1.40	2	1087	14.9	16.1	40929	26154	33364	63524	29805	47310
F1S6X1_PIG, TR:F1S6X1	30724.0	0.312338	1.24	2	4487	13.6	9.4	7261	5685	6868	3719	5436	6844
F1SHQ8_PIG, TR:F1SHQ8	50044.5	0.314326	-1.58	4	18619	16.7	9.2	23800	70079	37156	35097	79828	77412
F1RQ91_PIG, TR:F1RQ91	27504.2	0.316295	-1.10	4	2818	25.3	4.6	54694	67251	66056	75846	64096	68488
F1SMZ7_PIG, TR:F1SMZ7	60906.5	0.316452	-1.15	35	10106	67.7	0.9	450021	564050	477782	840493	547039	625101
F1S666_PIG, TR:F1S666	36439.5	0.316634	-2.35	2	611	9.5	4.5	1177	2271	1493	7919	1835	1882
F1SIK9_PIG, TR:F1SIK9	48584.6	0.318217	-1.53	3	1245	12.3	8.6	7186	34498	12510	18960	28614	35341
Q4TTS4_PIG, TR:Q4TTS4	21399.7	0.327148	-1.25	3	4265	27.2	5.4	76387	115365	108948	162142	113688	99817
F1RRT3_PIG, TR:F1RRT3	36820.0	0.328381	1.34	2	645	11.7	3.1	28195	33991	27409	12015	22267	32384
F1SHL0_PIG, TR:F1SHL0	51975.6	0.330108	1.53	3	808	13.2	12.8	10309	11983	8038	2217	9642	7967
F1SMB2_PIG, TR:F1SMB2	55350.0	0.331293	-1.24	2	1047	6.5	5.4	4337	4331	5050	6472	4083	6418
F1RJS2_PIG, TR:F1RJS2	51775.6	0.331992	-1.74	4	1042	12.3	6.2	17692	180550	75347	113404	166332	222437
F1SEX1_PIG, TR:F1SEX1	41748.1	0.332423	1.12	14	4263	53.1	10.6	177043	195580	159267	251305	177792	189707
F1RHJ2_PIG, TR:F1RHJ2	27104.5	0.33283	-1.32	4	1808	22.2	10.8	24034	49130	34622	55986	33160	52196
F1RX38_PIG, TR:F1RX38	56695.2	0.340884	-1.14	2	520	6.2	9.0	6216	6463	4694	7491	5976	6295
F1RFB5_PIG, TR:F1RFB5	37390.2	0.343232	-1.97	4	1834	18.4	14.2	17868	15850	15696	59668	15258	21839
F1SKI5_PIG, TR:F1SKI5	55918.1	0.343488	-1.28	2	652	6.2	4.5	14651	14471	15783	26207	14854	16398
COX41_PIG, SP:Q95283	11063.1	0.344761	-1.97	2	2096	24.7	24.4	3257	13234	1838	3650	14334	18114
F1RTQ5_PIG, TR:F1RTQ5	14270.6	0.345105	1.26	4	11658	26.0	9.9	6905	9561	7138	4268	7903	7361
F1S458_PIG, TR:F1S458	67847.9	0.346291	-1.19	5	939	12.6	7.2	43528	43305	37239	74349	39526	47823
F2Z5U0_PIG, TR:F2Z5U0	17964.9	0.348521	-1.18	5	25669	57.7	1.4	222942	235415	180022	269002	205006	206199
F1RKQ4_PIG, TR:F1RKQ4	59106.2	0.350428	-1.24	2	643	8.0	18.7	11558	17013	9481	14166	13185	19676
F2Z5N5_PIG, TR:F2Z5N5	17256.2	0.350907	-1.10	4	1334	13.1	3.5	27513	31760	30000	40848	30994	29018
F1SL01_PIG, TR:F1SL01	22447.6	0.353258	-1.76	2	2169	20.6	21.4	8684	9863	1731	15256	5385	15127
F2Z5L8_PIG, TR:F2Z5L8	32749.1	0.353986	1.88	3	3629	32.7	10.0	9260	19569	11150	10213	495	10512
F1RNU3_PIG, TR:F1RNU3	81483.2	0.355678	-1.48	4	1898	9.1	5.0	56072	67813	36035	117379	48705	67707
F1S543_PIG, TR:F1S543	36329.6	0.355857	-1.42	3	968	11.2	2.7	19598	21568	21920	45739	22648	21398
F1RWX8_PIG, TR:F1RWX8	120729.3	0.356412	1.38	16	3664	18.5	2.4	178697	136152	143528	216842	119872	158753
F1RIU3_PIG, TR:F1RIU3	57970.2	0.358189	-1.34	11	2114	30.9	2.7	90013	124888	128047	209094	132858	157575
F1SHW1_PIG, TR:F1SHW1	82761.7	0.358241	1.14	2	667	7.4	8.0	16860	16834	12373	13481	13068	13837
A5D9J3_PIG, TR:A5D9J3	80604.4	0.358792	-1.14	5	731	11.0	11.5	66628	84595	55257	83386	73728	87284
Q684M6_PIG, TR:Q684M6	44490.1	0.359017	-1.29	15	4806	40.8	2.7	209913	216475	190178	294058	156229	244568
F1RGK5_PIG, TR:F1RGK5	28922.4	0.362741	-3.70	5	9437	37.1	27.2	33171	17402	14919	156020	11429	67667
F1RG16_PIG, TR:F1RG16	45669.9	0.362825	-1.74	9	2544	37.9	8.1	47287	62824	65995	139451	57880	54348
TPM4_PIG, SP:P67937	28521.8	0.365014	-1.20	12	4779	45.6	6.6	291840	328331	241293	420773	256860	317947
F1RFI8_PIG, TR:F1RFI8	68936.7	0.365563	-1.22	2	2065	5.1	4.0	55604	44271	41044	71398	42607	57609
F1SKQ0_PIG, TR:F1SKQ0	72311.4	0.366672	-1.08	10	2111	17.5	2.2	112365	117154	100832	153783	106781	111607
F1SB63_PIG, TR:F1SB63	60354.5	0.367287	-1.08	15	2317	30.2	2.9	136552	176738	146252	217229	176277	216993
RS3_PIG, SP:Q0Z8U2	26688.3	0.367775	-1.43	5	4276	40.3	28.8	54637	97084	59896	65400	71760	145061
TERA_PIG, SP:P03974	89288.7	0.368363	-1.31	54	12967	77.0	7.6	827250	1006521	771327	1229719	742872	1201607
F2Z5M3_PIG, TR:F2Z5M3	16832.3	0.368665	-1.19	4	2612	29.2	4.1	27246	35361	33394	45957	35891	30605
F1SE73_PIG, TR:F1SE73	44868.2	0.368736	-1.11	3	3314	17.6	8.2	42832	48571	45080	56698	51795	42767
OSTF1_PIG, SP:Q8MJ49	23739.7	0.371142	-1.26	3	4597	13.1	8.2	33728	52157	27327	35843	46838	59457
F1S7L8_PIG, TR:F1S7L8	41557.0	0.373944	-1.31	4	691	13.6	16.8	11835	11487	8936	18971	11140	13243
F1SAC1_PIG, TR:F1SAC1	31576.5	0.375844	-1.13	2	2873	11.0	3.1	44641	64079	51851	64704	52500	63881
F1SUM3_PIG, TR:F1SUM3	24408.3	0.376543	-1.25	3	881	11.4	6.8	10988	23101	14034	20714	15688	23726
F1SPE8_PIG, TR:F1SPE8	54829.0	0.376587	-1.49	2	1241	9.0	11.6	7707	8789	1486	10100	7652	9008
CSK2B_PIG, SP:P67872	24942.4	0.378507	-1.33	3	3479	32.6	16.3	9021	28236	20318	21984	26214	28142
F1RGI7_PIG, TR:F1RGI7	95408.1	0.378697	-1.18	2	902	3.7	6.7	26056	27066	25524	38971	24931	28837
F2Z5B7_PIG, TR:F2Z5B7	9477.2	0.380602	1.12	2	2869	10.7	1.2	7547	10251	8087	7167	8004	7966
PIMT_PIG, SP:P80895	24646.4	0.380975	-1.23	3	3126	33.9	4.2	80585	88049	79424	113539	71493	120724
SAHH_PIG, SP:Q710C4	47694.0	0.381479	1.52	2	1031	3.5	6.2	45249	15795	31686	39752	8786	12424
CYTB_PIG, SP:Q29290	11130.6	0.382833	-3.71	3	4358	42.9	58.3	17187	14480	15980	142223	19133	15397
F1RUK8_PIG, TR:F1RUK8	50326.8	0.382874	-1.37	8	3354	32.8	6.3	141139	158675	65269	185664	143425	159439
F1SC51_PIG, TR:F1SC51	28573.3	0.384111	-1.38	15	10264	70.2	2.2	276385	308684	292377	543956	270808	302804
F1SID1_PIG, TR:F1SID1	96940.6	0.384772	1.59	2	357	4.5	14.6	4672	4076	2890	3848	306	3163
F1SNZ9_PIG, TR:F1SNZ9	5461.2	0.386729	-1.15	4	48818	74.5	8.7	19714	21454	20913	28495	18551	24762
F1SPP7_PIG, TR:F1SPP7	74281.2	0.396373	-1.64	2	491	8.3	10.3	40948	27186	582	47538	30130	35124
F1S530_PIG, TR:F1S530	34353.6	0.399453	-1.56	2	5044	17.5	7.0	29864	24523	26751	71369	25879	29360
F1RKD8_PIG, TR:F1RKD8	13995.5	0.399699	1.31	3	29449	61.8	13.8	168513	195931	133368	60114	161195	159031
F1S665_PIG, TR:F1S665	17170.3	0.399783	1.05	2	1070	15.9	6.9	7209	6258	6650	6125	6140	6834
F1S710_PIG, TR:F1S710	26495.2	0.400759	-1.37	2	1523	19.6	13.9	26357	41228	7987	32289	37043	34387
F1RWF5_PIG, TR:F1RWF5	30213.7	0.402709	1.09	6	4012	34.4	0.5	46854	48790	41789	45156	45317	50107
F1RVV0_PIG, TR:F1RVV0	18515.4	0.403888	-1.12	5	2852	37.3	5.8	102622	97284	85366	114108	86323	92266
F1SS24_PIG, TR:F1SS24	272274.8	0.405579	1.22	104	11305	54.7	4.2	2762655	3086001	4460810	3218870	2282457	3385276
F1RJ25_PIG, TR:F1RJ25	39678.1	0.406216	-1.11	14	34332	59.7	4.4	520133	631818	497084	647538	533887	701232
PALMD_PIG, SP:Q2MJV9	62307.7	0.409758	1.39	3	985	10.7	3.2	3064	4203	3586	757	3998	3069
GSTP1_PIG, SP:P80031	23497.0	0.4108	-1.17	8	14511	71.5	12.1	353019	490240	502838	606717	428393	473727
F1SLI3_PIG, TR:F1SLI3	116980.8	0.411506	-1.15	22	3085	37.4	2.2	265138	277834	192895	398786	235935	332914
F1SFQ5_PIG, TR:F1SFQ5	39596.4	0.414897	-1.12	18	3429	75.4	3.9	178484	269013	233718	366878	284303	313165
F1SNQ1_PIG, TR:F1SNQ1	194581.2	0.415667	-1.21	29	4613	26.5	6.3	190617	271303	199164	323963	231534	280509
F1RRY2_PIG, TR:F1RRY2	32603.0	0.415817	1.26	7	7831	27.6	4.8	143666	234947	233998	120392	185326	209144
PLAK_PIG, SP:Q8WNW3	81849.8	0.416954	-1.10	5	513	11.3	6.0	39718	43964	32433	38796	37645	47324
F2Z5K5_PIG, TR:F2Z5K5	49585.8	0.416957	-1.26	8	25937	45.7	4.0	22408	37318	28428	25412	31063	40836
F1SDE3_PIG, TR:F1SDE3	17231.8	0.418359	1.11	2	3555	21.4	2.3	21989	19955	17470	21558	16628	15151
F1S445_PIG, TR:F1S445	47788.3	0.418818	-1.33	3	2645	14.1	2.7	22398	24558	27987	47872	21454	30434
F1RFI1_PIG, TR:F1RFI1	49451.3	0.422557	-1.36	2	1062	4.9	17.6	14421	16258	15425	31141	13601	17775
F1SQT3_PIG, TR:F1SQT3	39809.5	0.42558	-1.10	3	3558	6.4	5.1	102472	127487	148219	145534	129139	142295
F1RZT0_PIG, TR:F1RZT0	48045.2	0.426011	1.19	7	2293	24.9	6.8	186766	148841	134656	151920	133321	155833
F1S994_PIG, TR:F1S994	20162.4	0.42686	1.46	2	1683	13.3	6.3	7727	7268	5962	7570	0	6791
F1RWT2_PIG, TR:F1RWT2	70797.0	0.4274	-1.14	26	5894	54.1	11.7	586904	661852	562397	858416	550898	739238
F1RPS8_PIG, TR:F1RPS8	57627.1	0.431183	-1.31	20	5671	39.0	1.8	360916	429231	384380	723144	435443	476775
PPIA_PIG, SP:P62936	17869.4	0.432413	-1.27	5	33576	82.3	9.7	19615	42779	27970	34300	51215	37556
F1ST99_PIG, TR:F1ST99	36393.7	0.434511	1.06	17	10792	62.8	3.0	434260	463772	434769	477674	431283	391853
F2Z5C7_PIG, TR:F2Z5C7	29944.9	0.437651	-1.16	17	4702	62.5	1.4	184888	291991	215546	396589	245869	315704
F2Z567_PIG, TR:F2Z567	28024.7	0.43977	-1.14	7	3694	37.4	1.4	134182	145539	126299	207042	141295	140659

F1S9K5_PIG, TR:F1S9K5	170137.3	0.441438	-1.20	6	1367	7.8	3.0	35839	31497	28775	54306	27255	37499
SRSF2_PIG, SP:Q06A98	25448.3	0.441441	1.33	3	7509	9.5	11.8	14300	22320	10216	14271	0	20901
F1SJR1_PIG, TR:F1SJR1	23576.3	0.44165	-1.15	3	4884	29.9	22.6	21403	36540	23096	31694	27569	33540
F2Z557_PIG, TR:F2Z557	70670.8	0.441727	-1.27	7	1153	14.5	25.5	85163	75720	92622	160779	96469	78938
F1SDN2_PIG, TR:F1SDN2	38295.0	0.442278	-1.16	4	2350	18.2	10.4	36677	35219	22936	44783	30350	35345
F2Z5Q2_PIG, TR:F2Z5Q2	17779.0	0.442324	-1.07	4	3094	22.3	3.3	168808	208621	174658	225857	194017	185490
F1SU59_PIG, TR:F1SU59	49541.8	0.445589	-1.20	2	1755	9.3	3.5	18149	24304	20614	32278	18272	25078
F1RS17_PIG, TR:F1RS17	21079.4	0.448064	-1.14	8	18764	50.3	5.4	362440	405099	366667	557245	375922	394944
RINI_PIG, SP:P10775	49023.3	0.448176	-1.13	26	6682	75.0	3.6	341421	353680	326853	549588	314336	442707
F2Z4Z1_PIG, TR:F2Z4Z1	28302.6	0.450718	1.46	11	8373	43.3	11.2	1114429	393180	816124	862015	431100	384728
F1RSG8_PIG, TR:F1RSG8	33835.4	0.451247	-1.19	2	1060	10.3	6.0	12209	12353	6555	14862	10059	12219
ACBP_PIG, SP:P12026	9896.3	0.452811	-1.26	2	776	41.4	4.5	21803	61346	45734	38826	63498	60588
F1RKY9_PIG, TR:F1RKY9	15785.8	0.455846	-1.23	2	1929	26.0	11.2	9504	25457	12615	15613	18141	24840
ENOB_PIG, SP:Q1KYT0	47130.1	0.457199	-1.34	4	7517	32.7	4.2	3186	3194	3098	6412	3221	3436
F1RJZ8_PIG, TR:F1RJZ8	16760.9	0.458763	-1.44	2	3644	37.7	12.2	591	1069	753	599	1110	1777
F1RQI3_PIG, TR:F1RQI3	126774.3	0.462009	1.17	5	1772	7.9	7.0	74750	47056	45099	57162	48174	45060
PGK2_PIG, SP:Q6RI85	44895.0	0.462367	-1.16	4	5442	19.2	9.9	64891	108002	76035	75964	110674	100817
ADH1_YEAST, SP:P00330	36849.2	0.463154	1.12	25	10647	72.1	78.9	1072449	1109716	1177564	1243373	913101	971368
MX1_PIG, SP:P27594	75587.4	0.464301	1.10	3	2994	7.4	12.5	38178	42310	38050	43088	28991	36019
ACTB_PIG, SP:Q6QAQ1	41736.7	0.469566	-1.09	59	116678	99.5	0.7	8355553	8578460	8458867	10883000	8653774	11213000
F1ST86_PIG, TR:F1ST86	23237.4	0.477607	1.30	6	28428	24.5	12.3	80949	100557	72694	96429	13043	89182
MT2A_PIG, SP:P79379	5970.1	0.477862	1.12	2	45325	52.5	11.1	2035	2101	1950	2452	1478	1487
F1RPN9_PIG, TR:F1RPN9	66329.7	0.478612	-1.27	2	865	10.2	14.6	6184	12324	8390	7988	9692	16451
ENO1_YEAST, SP:P00924	46816.1	0.48124	1.28	4	1000	13.3	14.2	10016	8719	11555	12927	1037	9590
F1SP06_PIG, TR:F1SP06	100328.4	0.481732	-1.14	11	1244	19.5	4.7	191895	177830	121658	206723	133310	164548
F1S663_PIG, TR:F1S663	177248.1	0.485059	-1.43	3	492	4.2	13.7	18594	9123	6753	27728	9701	11772
PURA2_PIG, SP:A4Z6H1	50140.3	0.486719	-1.34	2	1035	7.5	14.5	2621	14395	8730	6746	11704	16003
F1RKG8_PIG, TR:F1RKG8	20971.7	0.487173	-1.60	7	4489	51.3	7.6	270859	190984	150715	536777	169286	249656
F1SA50_PIG, TR:F1SA50	74943.4	0.487384	1.11	25	2624	54.7	6.5	522660	488980	411403	556441	395050	410462
F2Z5H8_PIG, TR:F2Z5H8	17699.0	0.488746	-1.08	3	1720	21.6	17.3	11171	14535	7708	17034	0	19199
F1RPH0_PIG, TR:F1RPH0	41388.8	0.49039	-1.11	10	37982	63.2	3.1	167452	229235	172124	187814	197476	237077
F1SLA9_PIG, TR:F1SLA9	20314.0	0.497825	-1.34	6	4670	43.7	4.5	33661	37430	32517	61421	25724	47464
F1S1N1_PIG, TR:F1S1N1	40941.9	0.499572	1.26	2	1423	16.8	10.8	17091	37198	27572	23070	23742	18120
F1RX01_PIG, TR:F1RX01	59756.8	0.500881	-1.21	2	1161	6.8	20.9	23493	36254	25981	47343	24892	31393
F1S4Y6_PIG, TR:F1S4Y6	11346.0	0.502205	1.10	2	4522	16.2	10.2	21026	25513	21725	15916	23166	23038
F2Z5W4_PIG, TR:F2Z5W4	36926.5	0.50579	-1.11	2	1254	7.5	7.4	7469	8112	6342	9411	6511	8383
F1SIH7_PIG, TR:F1SIH7	63198.6	0.510247	1.11	10	6346	36.7	5.8	69908	92173	62021	90737	74655	78860
F1S593_PIG, TR:F1S593	52263.7	0.5125	-1.23	2	719	9.7	14.8	3218	5633	3541	6076	0	9164
F1RY84_PIG, TR:F1RY84	56882.0	0.512787	1.26	11	5945	29.6	14.2	57040	71466	60012	62690	19597	58769
F1RZK9_PIG, TR:F1RZK9	32269.7	0.513544	-1.25	2	1330	24.6	35.4	7974	4581	4049	8278	0	12412
F1SN31_PIG, TR:F1SN31	50896.4	0.51506	-1.18	5	2993	18.6	7.8	32293	49357	17144	43763	42992	35829
F1S269_PIG, TR:F1S269	40192.1	0.517154	-1.08	2	936	7.1	9.4	17091	6406	0	18398	5427	1624
F1S6R1_PIG, TR:F1S6R1	18606.9	0.517295	-1.68	2	1271	25.6	15.4	6885	5683	6174	19114	4293	8116
F2Z5V6_PIG, TR:F2Z5V6	19865.2	0.518543	-1.31	5	6959	58.1	32.5	18457	76074	18977	22753	58263	64939
F1RZH3_PIG, TR:F1RZH3	56197.6	0.525065	1.29	2	577	10.4	32.7	21822	7011	19610	26773	6577	4135
F2Z5D3_PIG, TR:F2Z5D3	14395.3	0.52874	-1.09	2	9369	30.7	7.8	29392	42253	26146	34899	34643	37312
F1SHL7_PIG, TR:F1SHL7	57190.1	0.529218	-1.13	34	61625	78.7	5.1	1794908	2305336	1817357	2678646	1858935	2612460
F1SL58_PIG, TR:F1SL58	45072.1	0.529446	-1.12	3	1073	10.3	2.5	17356	20552	20189	27729	18640	18889
STAT1_PIG, SP:Q764M5	88166.7	0.532017	-1.45	2	545	3.2	12.5	7975	8365	4547	17527	0	12826
F1SPB9_PIG, TR:F1SPB9	17208.8	0.535215	-1.13	2	3154	18.5	0.8	16520	20089	13271	14339	21666	20240
F1RKK2_PIG, TR:F1RKK2	130478.0	0.538159	-1.10	2	501	4.1	4.6	5896	7400	6076	8639	6487	6139
F1S585_PIG, TR:F1S585	88716.2	0.540069	1.09	8	1687	15.6	16.0	165788	170881	167014	235042	129440	133731
F1SA52_PIG, TR:F1SA52	85010.4	0.54057	-1.26	2	497	5.1	11.3	23523	19792	5568	25036	12115	24656
GTR1_PIG, SP:P20303	49777.6	0.540829	1.15	3	993	6.0	16.7	55740	62837	60836	57520	26144	72101
F1SS65_PIG, TR:F1SS65	223212.1	0.550231	-1.07	6	8185	4.3	9.3	21856	26060	21506	24936	21210	22112
COMT_PIG, SP:Q99028	20586.8	0.556499	-1.05	2	2153	21.0	8.9	7471	8077	6416	7765	7083	8240
F1SNH3_PIG, TR:F1SNH3	63036.7	0.556726	-1.48	9	1045	24.8	6.6	44786	56956	44414	98547	28163	52989
F1SLG5_PIG, TR:F1SLG5	34499.4	0.557627	-1.09	4	31063	26.9	4.9	37128	39254	25872	42792	33895	34897
F2Q9A3_PIG, TR:F2Q9A3	17869.4	0.558405	1.05	10	45272	85.4	5.8	875069	822854	716916	772436	735500	864044
F1SMV6_PIG, TR:F1SMV6	39542.8	0.568014	-1.08	11	5701	33.4	8.8	190367	257662	181364	264395	182961	288683
D4N5N1_PIG, TR:D4N5N1	48287.8	0.574526	-1.19	16	6526	49.9	3.2	312818	686108	359423	460016	523493	758819
F1SA98_PIG, TR:F1SA98	24865.9	0.576522	-1.35	2	1053	11.8	30.4	20162	28224	23252	53912	20177	22746
F1RJT3_PIG, TR:F1RJT3	62321.6	0.577875	-1.07	30	7205	74.5	3.9	564206	593235	488329	673623	519555	581086
F1S0D9_PIG, TR:F1S0D9	57143.3	0.578962	-1.16	2	1319	7.0	16.2	30169	30349	31744	48187	32751	26174
F1RJT0_PIG, TR:F1RJT0	46971.8	0.579436	1.08	3	1115	9.5	2.9	12075	17788	12595	20881	1290	17120
F1S4X8_PIG, TR:F1S4X8	103742.9	0.581492	1.10	2	8109	3.2	2.8	1997	2351	2543	1404	2723	2118
F1SU97_PIG, TR:F1SU97	57990.4	0.591442	1.24	3	1710	9.2	7.6	33217	13385	19842	26687	13997	12788
F1SLF6_PIG, TR:F1SLF6	59342.6	0.591777	-1.09	5	1347	18.3	12.6	47528	44090	43948	57950	37569	52913
ATP5I_PIG, SP:Q9MYT8	8231.6	0.592319	1.13	2	13075	21.1	6.0	34326	33716	24630	35099	13840	32765
RL3_PIG, SP:Q29293	14792.4	0.593858	-1.57	2	2544	16.9	27.5	9435	7332	10741	27468	8377	7239
F2Z5D2_PIG, TR:F2Z5D2	47371.2	0.598863	1.12	20	6480	69.9	3.6	114038	181030	138927	151269	168826	190982
F1RLQ2_PIG, TR:F1RLQ2	74232.5	0.599658	1.17	17	8117	48.9	4.8	504778	480033	435240	594236	427683	441366
F2Z543_PIG, TR:F2Z543	29804.1	0.603061	-1.54	2	2382	17.3	21.5	3810	3645	4820	10339	2328	6249
F6PT10_PIG, TR:F6PT10	11329.6	0.605874	-1.13	7	33343	72.5	23.7	203137	289426	257321	383008	258372	235976
VINC_PIG, SP:P26234	123944.5	0.606272	-1.28	22	2851	37.3	5.1	183016	199714	184585	411100	210125	257797
F1RJ93_PIG, TR:F1RJ93	23302.5	0.610647	-1.09	24	8596	90.9	8.5	653783	1233091	861153	1518689	1050269	1086290
F1SQ01_PIG, TR:F1SQ01	30555.0	0.612423	-1.10	2	975	11.8	8.6	8116	14116	9685	9398	11726	14039
F1S2B6_PIG, TR:F1S2B6	41775.3	0.615232	1.14	2	987	8.9	7.5	12958	8484	15342	12355	10988	8879
F1SJY6_PIG, TR:F1SJY6	104839.5	0.621908	-1.28	4	1167	9.3	8.8	29862	43831	31795	56341	24364	48549
F1RVA0_PIG, TR:F1RVA0	15223.8	0.623258	-1.05	2	22281	36.0	2.6	16547	21944	17718	21666	18532	18807
F1S3P6_PIG, TR:F1S3P6	37934.2	0.626924	1.08	13	12865	62.8	3.1	298738	289801	214371	305379	287815	253060
F1SFH7_PIG, TR:F1SFH7	24187.1	0.628117	-1.07	7	1574	54.7	0.3	47663	47563	43958	61859	43557	46870
F1S5N0_PIG, TR:F1S5N0	69853.8	0.629733	-1.11	2	815	5.5	2.6	15290	16521	13858	21104	12673	16913
AATC_PIG, SP:P00503	46474.8	0.631886	-1.14	2	716	9.0	11.2	16686	28407	18986	16698	24905	31767
F1SRK6_PIG, TR:F1SRK6	56025.9	0.634224	-1.20	2	3176	11.9	9.8	61327	54144	42348	89863	39030	61140
F1RVC9_PIG, TR:F1RVC9	23029.6	0.634592	-1.10	4	2337	18.8	21.8	55395	73112	60335	80019	52011	78951

IPKG_PIG, SP:Q7YQJ4	7912.4	0.635336	1.06	3	3342	73.7	3.1	11607	12959	12228	8867	11461	14335
F1SM78_PIG, TR:F1SM78	15258.0	0.639017	-1.57	3	8568	39.8	56.0	20968	29517	28837	78882	236	45621
F2Z5S8_PIG, TR:F2Z5S8	49924.4	0.640061	1.11	5	19929	54.0	21.0	52031	108400	86383	23350	93978	113749
F1RX16_PIG, TR:F1RX16	74576.8	0.642316	-1.20	6	909	9.9	1.7	63722	75457	67363	110344	69414	75761
F1SRV9_PIG, TR:F1SRV9	61137.4	0.643797	1.09	9	1873	36.3	5.3	58169	69438	74030	82878	63601	59327
ANXA2_PIG, SP:P19620	38541.7	0.64804	1.09	9	10593	58.7	9.8	336894	449145	338503	397041	179798	477129
STMN1_PIG, SP:Q6DUB7	17302.5	0.650218	-1.36	3	3140	21.5	4.2	34300	32561	30090	74746	23285	33383
F1RQ90_PIG, TR:F1RQ90	42274.8	0.651934	-1.06	2	3569	8.2	8.7	23891	41282	34997	34221	36277	35906
F1RII4_PIG, TR:F1RII4	14419.9	0.654535	-1.07	12	17740	80.4	10.4	330193	416966	290128	508620	327603	439365
F1RKM0_PIG, TR:F1RKM0	66559.4	0.657297	1.12	11	2198	22.2	10.7	90877	110482	73319	116599	37586	101172
F1SCY1_PIG, TR:F1SCY1	54839.5	0.657603	-1.06	2	3982	5.7	3.9	32290	37376	30392	40353	29015	37117
G6PI_PIG, SP:P08059	63126.1	0.662771	-1.06	6	2432	20.3	9.7	88361	142908	129805	130374	128466	121379
F1RRJ5_PIG, TR:F1RRJ5	27724.0	0.663487	-1.13	2	1863	17.3	6.5	22256	35492	23486	36886	20244	34975
ESTD_PIG, SP:Q9GJT2	31481.8	0.663681	-1.06	3	1536	23.4	15.5	28114	46022	38745	44499	38841	36428
F2Z546_PIG, TR:F2Z546	23247.7	0.664079	-1.03	4	6243	22.7	1.7	55710	63116	51644	61022	57353	56080
F1SU03_PIG, TR:F1SU03	282269.0	0.666558	1.13	2	371	1.5	12.4	6006	4799	3240	4339	3547	4571
F1SIP0_PIG, TR:F1SIP0	41152.0	0.667249	-1.11	10	1964	38.5	1.0	117271	154192	121849	192454	134244	137683
F1SA20_PIG, TR:F1SA20	98673.8	0.667257	-1.08	32	4958	54.5	12.0	377800	489003	452129	825042	466133	568361
F1SG37_PIG, TR:F1SG37	55978.0	0.669137	-1.11	5	3059	20.6	11.5	48948	45523	37666	42915	37527	58894
F1RN65_PIG, TR:F1RN65	9442.5	0.66972	-1.15	2	11378	61.5	34.4	7818	12306	10383	7398	14711	13014
F1RGJ2_PIG, TR:F1RGJ2	39329.9	0.671993	1.06	3	3186	10.9	10.0	21939	26924	19929	17400	23604	23790
F2Z5L7_PIG, TR:F2Z5L7	29555.6	0.674263	1.07	11	2828	53.2	3.1	105728	91124	67715	113845	83323	102042
PRDX6_PIG, SP:Q9TSX9	25036.9	0.674402	-1.10	2	1802	14.7	3.8	27549	23625	15165	31303	18613	23286
F1SQ46_PIG, TR:F1SQ46	25465.0	0.676281	-1.09	6	7055	45.5	8.4	45405	58770	39175	60083	38543	55302
F1S0R2_PIG, TR:F1S0R2	43582.2	0.676662	1.03	5	2660	12.5	1.8	134222	114002	115571	138920	106113	113659
F1RFY2_PIG, TR:F1RFY2	47114.0	0.681612	1.07	5	16637	32.3	12.5	75159	76026	98369	60140	81721	99464
ACON_PIG, SP:P16276	85760.9	0.685103	-1.19	2	1298	6.3	10.5	1601	3486	783	3902	1898	1214
CTGF_PIG, SP:O19113	38007.3	0.689116	1.05	5	4749	49.3	2.9	61017	65409	53880	49761	70155	54449
F2Z5B2_PIG, TR:F2Z5B2	49953.1	0.706928	-1.06	6	14117	53.0	1.2	42708	42000	29661	35265	35320	47812
F1SPG0_PIG, TR:F1SPG0	23539.9	0.706988	-1.06	3	1456	12.1	14.0	43458	36736	34311	48143	33076	40302
LMNA_PIG, SP:Q3ZD69	74218.5	0.713994	-1.19	21	6839	54.5	6.8	559828	702699	568912	1012137	472572	775261
GPX1_PIG, SP:Q8MJ14	22590.8	0.714752	1.03	9	3103	65.0	3.8	103721	103716	102455	152366	102148	108461
F1SV90_PIG, TR:F1SV90	29308.1	0.727464	1.04	4	1510	23.9	3.4	52493	45597	48365	63161	37691	38328
F1RU18_PIG, TR:F1RU18	18341.4	0.728562	1.04	3	36676	48.5	10.3	32433	39424	36974	25981	36122	42154
MYH7_PIG, SP:P79293	223298.4	0.740918	-1.05	2	8109	1.1	15.1	1897	3382	1637	2993	2132	2156
Q06A94_PIG, TR:Q06A94	34196.2	0.74281	1.04	9	6510	52.2	6.4	268546	236786	238987	358356	207199	199041
A0PFK5_PIG, TR:A0PFK5	33016.9	0.745397	1.22	3	2730	22.4	40.1	887776	294565	411604	619376	285744	400775
F1RZE7_PIG, TR:F1RZE7	38725.0	0.753555	-1.08	6	2061	27.2	11.3	117275	177878	126169	197896	118397	152581
F1S2E2_PIG, TR:F1S2E2	54143.5	0.755697	-1.14	5	1786	12.9	9.1	32073	34491	25781	48150	23416	39716
A5GFU0_PIG, TR:A5GFU0	107475.8	0.758424	1.15	2	3935	5.4	9.4	2352	781	1594	2199	1183	716
F2Z4Y8_PIG, TR:F2Z4Y8	18430.7	0.769643	-1.20	8	8595	47.5	9.4	92458	93244	97278	158562	96647	90728
F1SHC1_PIG, TR:F1SHC1	49927.4	0.769737	1.06	4	12843	46.1	3.4	22744	39912	31348	16410	42831	36957
F1S596_PIG, TR:F1S596	60549.4	0.773019	-1.09	5	1646	14.7	8.1	34618	34264	31682	42230	33490	28655
F1SCY2_PIG, TR:F1SCY2	56088.8	0.776075	-1.03	12	9691	40.8	8.4	45436	79569	24025	57208	36040	67075
F1SQW0_PIG, TR:F1SQW0	48113.9	0.781906	1.06	4	1708	18.2	8.5	39054	42238	24047	46938	16281	37091
F2Z5F5_PIG, TR:F2Z5F5	24205.2	0.782368	-1.08	14	8373	57.7	36.7	368707	273340	242732	412919	287397	309888
MDHC_PIG, SP:P11708	36454.2	0.783193	-1.03	16	9124	51.5	2.7	313855	385675	288834	371455	354511	354742
F2Z5C1_PIG, TR:F2Z5C1	36134.0	0.78803	-1.28	30	23337	77.9	4.0	838188	1802452	978757	1909299	1892765	2471991
F1S415_PIG, TR:F1S415	61477.4	0.789151	-1.08	2	612	7.3	18.3	5768	5595	5584	8254	5296	4802
F1RK98_PIG, TR:F1RK98	49956.5	0.78959	-1.04	4	18346	39.3	27.0	1665	3446	2742	2688	3392	2412
F2Z5N0_PIG, TR:F2Z5N0	27399.5	0.789667	1.03	9	4584	40.2	6.8	88766	132097	101624	88209	130059	144225
F1SPP8_PIG, TR:F1SPP8	56993.4	0.798858	1.10	12	2437	31.6	19.0	107877	72769	84046	171940	82771	95509
F1S189_PIG, TR:F1S189	29998.8	0.805748	1.05	2	2810	15.2	4.5	449	701	641	565	439	699
F1S6R7_PIG, TR:F1S6R7	59694.1	0.809118	1.01	5	2061	14.0	5.3	252299	246944	247819	271412	236899	243905
COPB_PIG, SP:D2SW95	107123.3	0.813089	-1.05	3	1808	6.5	2.4	60728	58798	49619	63717	45154	68870
F2Z576_PIG, TR:F2Z576	15404.1	0.818458	-1.03	2	13356	47.1	18.3	14700	22467	23256	20247	23667	18161
F1S1N3_PIG, TR:F1S1N3	44052.6	0.819469	-1.15	7	4303	30.8	6.1	49163	95258	75906	101436	45020	112455
F1SJQ5_PIG, TR:F1SJQ5	34133.7	0.820155	-1.02	4	9435	39.2	24.0	16455	46098	29350	23853	39888	29624
TBA1A_PIG, SP:P02550	50068.5	0.821513	1.09	9	20239	54.5	2.9	28005	29522	29254	19537	25333	21453
F1S879_PIG, TR:F1S879	16355.6	0.822424	-1.39	3	9756	23.5	16.6	5540	3483	12993	21948	4123	4489
F2Z5R7_PIG, TR:F2Z5R7	6676.8	0.822967	-1.07	2	5233	33.9	10.1	25171	25066	19611	33714	19437	21668
F1SU06_PIG, TR:F1SU06	61033.9	0.824636	-1.06	2	506	6.7	4.4	30441	26502	25954	36645	21954	29215
F1RFB4_PIG, TR:F1RFB4	36211.6	0.828274	-1.00	7	1482	36.1	7.8	59874	34106	17651	64231	24454	35411
ODO2_PIG, SP:Q9N0F1	48976.6	0.829024	-1.08	4	1960	7.5	5.4	24740	45980	25139	43651	15285	41794
LMCD1_PIG, SP:Q5PXT2	40659.5	0.829295	-1.10	3	1444	15.7	1.8	17654	19490	20061	30058	16518	16165
MYH1_PIG, SP:Q9TV61	223173.3	0.847158	-1.03	2	8132	1.7	9.3	15708	15019	13873	15527	12662	17809
F1RP17_PIG, TR:F1RP17	63728.4	0.847604	-1.03	23	4775	57.5	2.7	373855	503500	588263	686927	500036	563761
F1RJK4_PIG, TR:F1RJK4	61066.0	0.848182	1.02	7	2760	22.4	10.6	99311	93447	81020	119491	90489	84657
F1RVH7_PIG, TR:F1RVH7	29102.3	0.848783	1.05	2	5128	11.7	14.4	26003	22257	15291	25083	15598	19910
F1SBT6_PIG, TR:F1SBT6	73993.3	0.850464	-1.01	9	3347	20.8	4.6	210395	175837	176319	268140	189050	188198
F1RM74_PIG, TR:F1RM74	39359.5	0.860323	-1.04	11	10699	59.7	4.7	28532	40109	33777	35044	39517	31213
F1RGG1_PIG, TR:F1RGG1	17343.9	0.86745	1.02	2	2846	19.7	21.8	17723	9535	12440	20253	10482	8331
ACADM_PIG, SP:P41367	46485.2	0.870326	-1.22	2	1194	8.8	19.9	11552	12250	9784	21717	6003	13092
F1ST81_PIG, TR:F1ST81	48845.1	0.880213	-1.07	11	2834	34.4	6.9	107116	69241	78889	137863	58402	76564
ATP5J_PIG, SP:P13618	8930.0	0.881524	1.03	2	8996	30.3	3.3	4410	19256	15812	7336	14331	16482
EF1G_PIG, SP:Q29387	49624.1	0.883495	1.06	2	3635	12.7	40.1	604	1297	592	422	818	1120
F1RQU9_PIG, TR:F1RQU9	14383.7	0.884797	-1.04	2	9171	8.3	3.4	51608	56378	43474	67710	49800	40598
F1SS68_PIG, TR:F1SS68	222850.8	0.884836	1.02	4	8124	3.3	14.6	169903	79839	39001	145013	79462	58608
F1SV88_PIG, TR:F1SV88	20040.4	0.892545	-1.14	7	12278	53.8	0.2	32819	27413	22659	36972	14234	35410
F1SRS2_PIG, TR:F1SRS2	25895.3	0.898652	1.16	9	36365	86.6	3.3	228217	604741	328791	293173	341649	336005
F1SU37_PIG, TR:F1SU37	123816.3	0.901723	1.00	11	1921	28.3	9.5	152812	210545	214656	219704	221625	242557
F1RPW9_PIG, TR:F1RPW9	50311.9	0.905875	1.00	13	4948	41.0	5.4	253488	334372	270039	420278	193128	313473
F1RPW8_PIG, TR:F1RPW8	25134.5	0.90918	-1.07	2	1628	12.0	15.6	19874	11885	16193	25392	11699	14255
KCY_PIG, SP:Q29561	22279.4	0.916329	1.00	5	5129	25.5	2.9	81114	77667	76119	95344	69715	86629
F1RL02_PIG, TR:F1RL02	37442.2	0.92373	-1.06	3	2552	13.1	0.7	62574	48730	71383	89586	44326	58934
F1S798_PIG, TR:F1S798	41052.9	0.924979	-1.01	6	2586	25.4	4.4	102823	105293	90711	108921	77944	114207

F1RKU0_PIG, TR:F1RKU0	39631.8	0.927273	-1.03	2	1105	5.5	4.5	11456	11536	11035	16547	7085	11529
RS17_PIG, SP:Q6QAP7	15524.1	0.927629	1.01	5	8524	53.3	12.5	48538	69637	39368	32298	51857	66058
F1SFZ8_PIG, TR:F1SFZ8	270393.8	0.927728	1.01	37	1233	25.4	6.3	559611	474795	510568	697020	449619	573905
F1RS15_PIG, TR:F1RS15	83280.2	0.927809	1.00	3	1413	7.8	3.9	26172	29885	26735	30597	21917	30116
F1SKJ5_PIG, TR:F1SKJ5	63960.7	0.928121	-1.02	4	1055	17.2	7.7	57191	63290	52148	77916	48237	56019
PDXK_PIG, SP:O46560	35613.7	0.929923	-1.06	15	4318	69.6	1.1	253649	275080	247630	275007	228119	293974
F1RUI1_PIG, TR:F1RUI1	27159.3	0.938889	-1.04	3	2699	18.4	16.1	36496	116023	61236	32869	97365	91247
F1SG65_PIG, TR:F1SG65	77973.8	0.942113	-1.11	2	1397	5.4	10.4	9322	11294	8673	16498	4323	11590
F1RMQ2_PIG, TR:F1RMQ2	15730.5	0.942904	-1.01	2	3639	19.7	6.9	49826	63905	48270	45492	60288	57660
F1S8Y5_PIG, TR:F1S8Y5	28803.9	0.945766	1.01	21	18009	72.0	2.2	889526	808554	732855	998428	752776	873462
F1SB09_PIG, TR:F1SB09	24756.6	0.946751	1.04	2	2692	20.0	13.5	19928	23513	9866	15996	15843	19394
HS71A_PIG, SP:P34930	70083.3	0.947473	-1.00	12	4892	41.8	5.0	228507	292061	324323	343785	283828	258154
C0MHR2_PIG, TR:C0MHR2	191614.6	0.952524	1.01	39	2309	27.6	4.1	775146	970222	768553	1229205	947359	1045767
F1S187_PIG, TR:F1S187	62474.5	0.953238	-1.08	2	651	3.2	17.6	6366	5843	5684	10453	3848	4952
D3K5N7_PIG, TR:D3K5N7	36134.9	0.962163	-1.01	7	2667	33.4	1.1	87229	102078	84803	120012	78473	88175
TPM3_PIG, SP:A1XQV4	33058.2	0.962499	1.00	6	6725	16.5	10.4	155967	156021	145241	133650	134286	146685
DESM_PIG, SP:P02540	53629.0	0.963258	-1.01	3	2015	7.9	9.5	13054	9004	8483	12821	7820	10237
F2Z4X8_PIG, TR:F2Z4X8	49959.5	0.967812	-1.04	4	24943	22.0	18.7	4768	5131	2887	2204	5395	5856
F1SJJ5_PIG, TR:F1SJJ5	49294.3	0.968907	1.00	13	2782	39.1	7.3	146402	110705	104118	192955	107131	90514
SRSF1_PIG, SP:Q3YLA6	27744.6	0.969669	-1.09	6	2432	24.2	11.3	154822	82135	113482	211696	113429	66224
F1SLT7_PIG, TR:F1SLT7	15223.4	0.971723	-1.02	12	14105	85.2	6.9	308112	349204	304284	333904	318897	396037
F1RJZ9_PIG, TR:F1RJZ9	14965.7	0.974975	-1.00	3	2090	29.5	3.2	5252	8190	8097	5607	7135	8879
FUMH_PIG, SP:P10173	50009.3	0.976158	-1.01	2	924	7.9	10.4	27181	33434	25658	20359	31737	35434
F1SJS8_PIG, TR:F1SJS8	18312.9	0.985484	-1.02	2	1850	15.2	8.8	43395	46228	36603	56081	34571	37504
F1S2D1_PIG, TR:F1S2D1	14099.4	0.988508	-1.02	2	5337	11.7	8.0	22953	21685	17945	28541	17362	18174
F1SJE2_PIG, TR:F1SJE2	129897.7	0.98904	-1.01	4	404	4.8	2.6	52218	49569	40015	61338	39988	50179
F1S087_PIG, TR:F1S087	49145.9	0.991257	-1.08	2	1629	9.8	11.3	8656	10508	7995	12109	4782	12409
F1RHM8_PIG, TR:F1RHM8	23741.5	0.991606	1.01	9	8055	63.1	4.5	203821	277499	169211	305105	163121	263564
F1SUN8_PIG, TR:F1SUN8	23226.2	0.993145	-1.36	3	4966	38.8	65.1	23008	2948	10139	41034	3811	4281
ALDR_PIG, SP:P80276	35868.3	0.994131	1.00	3	1582	12.7	7.2	48147	73551	58773	47231	65993	66514
TBB_PIG, SP:P02554	49861.0	0.994517	1.01	9	23524	42.0	4.6	283232	231949	219103	284118	199617	268737
F1RGJ3_PIG, TR:F1RGJ3	73824.6	0.997345	-1.00	17	2688	33.0	1.6	378540	411923	357673	546076	367058	414773

Table S3. Supp.Info: List of peptides used in the validation experiment. WT:PrP 106-126 treated PBEC; NT, Control.

ProteinName	ModifiedSequence	nt_1	nt_2	nt_3	wt_1	wt_2	wt_3	Ratio[1] (NT/WT)	T-Test P Value
F1RFY1	SSFFVNGLTLGGQK	22315922	124319507.1	156299271.9	146735985.7	288807416.6	298704526.7	0.41	0.08902
F1RFY1	TLVLLMGK	89085.456	2953567.06	3540280.104	5888295.792	10924088.33	9167836.546	0.25	0.02777
F1RGA9	NADPILISLK	272845.356	1377269.654	1176948.146	3216022.815	1465876.784	1963041.351	0.43	0.12137
F1RGA9	QLALWNPK	551241.583	2561761.926	2992154.969	4849176.417	4974760.658	3521701.492	0.46	0.06409
F1RMJ4	LQQTYAALNSK	5462.4163	56348.37085	58952.51085	100019.7657	139595.6877	101466.7182	0.35	0.03129
F1RMJ4	LTELGTVDPK	17323.6372	197472.471	226256.871	395655.9328	535229.3613	410919.9507	0.33	0.02411
F1RTN3	IGFPWSEIR	2540010.05	33748211.19	32595510.44	79948324.15	94904512.6	90727726.89	0.26	0.01264
F1S073	LMVALAK	13334383.9	59177265.14	67254287.78	96628574.81	85526477.21	79619639.62	0.53	0.12693
F1S073	TNQELQEINR	824184.347	7011970.905	7337312.248	12713437.51	19248427.28	11349084.47	0.35	0.04498
F1SGJ5	SGC[+57]IVDNLAEFTVDPK	22041318.8	98149599.19	66019901.55	140073387.4	148668612.9	142326537.4	0.43	0.06405
F1SNF3	LYTLLC[+57]QAAK	75297.4741	288901.9431	695960.8123	1376587.32	1691229.017	1474533.188	0.23	0.01105
F1SQN1	LGGTIDDC[+57]ELVEGLVLTQK	704438.694	2992975.416	2563152.299	3062588.976	3255410.547	3675779.364	0.63	0.21349
F1SQN1	AVADAIR	351.4256	3051.96145	8133.1249	8393.13305	7987.8136	4026.1281	0.57	0.34248
F6PT10_PIG	MAPYQGPDAVPGALDYK	40250586.2	159014954.6	96083696.89	98708720.54	169842839.2	208036967.1	0.62	0.26778
F6PT10_PIG	ETTDTDTADQVIASFK	1036.4532	5374.29405	10611.7236	23649.4681	37696.88495	32266.1124	0.18	0.00939
TBA1B_PIG	YMAC[+57]C[+57]LLYR	6180.6108	51990.5201	73688.58125	226835.3941	209789.685	144571.4998	0.23	0.01066
TBA1B_PIG	SIQFVDWC[+57]PTGFK	6953759.72	72053809.4	83748168.94	153215185.7	177920177.5	194273438.3	0.31	0.02106

[1]Ratio of PrP/control was determined based on the average protein measurement in each group, which also passed the filtering criteria listed in Materials and Methods section.
